# Supplementary material for: Low-Affinity/High-Selectivity Dopamine Transport Inhibition Sufficient to Rescue Cognitive Functions in the Aging Rat
Source: Biomolecules. 2023 Mar 3;13(3):467. doi: 10.3390/biom13030467 (PMC10046369; doi:10.3390/biom13030467)
Supplement: Supplementary file 1 [file biomolecules-13-00467-s001.zip › biomolecules-2185640-supplementary.pdf]

Supporting Information for:

Low-affinity/high selectivity dopamine transport inhibition sufficient to rescue cognitive functions in the aging rat

Jana Lubec [1,†,‡], Ahmed M. Hussein [1,2,3,†], Predrag Kalaba [2,†], Daniel Daba Feyissa [2], Edgar Arias-Sandoval [4], Anita Cybulska-Klosowicz [5], Mekite Bezu [2], Tamara Stojanovic [1], Volker Korz [1], Jovana Malikovic [2], Nilima Y. Aher [2], Martin Zehl [6], Vladimir Dragacevic [1,2], Johann Jakob Leban [1], Claudia Sagheddu [7], Judith Wackerlig [2], Marco Pistis [7,8], Merce Correa [4,9], Thierry Langer [2], Ernst Urban [2], Harald Höger [10] and Gert Lubec [1,*]

1 Programme for Proteomics, Paracelsus Medical University, 5020 Salzburg, Austria; jana.aradska@gmail.com (J.L.); hamadaz81@gmail.com (A.M.H.); tamara.stojanovich@gmail.com (T.S.); volkerkorz@arcor.de (V.K.); dragacevicvladimir@gmail.com (V.D.); j-e-leban@outlook.com (J.J.L.)

2 Department of Pharmaceutical Sciences, Division of Pharmaceutical Chemistry, Faculty of Life Sciences, University of Vienna, 1090 Vienna, Austria; peja87.bgd@gmail.com (P.K.); daniel.daba@ju.edu.et (D.D.F.); mekiteb@yahoo.com (M.B.); malikovicjovana@gmail.com (J.M.); ahernilima12@gmail.com (N.Y.A.); judith.wackerlig@univie.ac.at (J.W.); thierry.langer@univie.ac.at (T.L.); ernst.urban@univie.ac.at (E.U.)

3 Department of Zoology, Faculty of Science, Al-Azhar University, 71524 Assiut, Egypt

4 Department of Psychobiology, Universitat Jaume I, 12006 Castelló, Spain; ariasdes@uji.es (E.A.-S.); correa@uji.es (M.C.)

5 Neurobiology of Emotions Laboratory, Nencki Institute of Experimental Biology, 02093 Warsaw, Poland; a.cybulska@nencki.edu.pl

6 Department of Analytical Chemistry, Faculty of Chemistry, University of Vienna, 1090 Vienna, Austria; martin.zehl@univie.ac.at

7 Department of Biomedical Sciences, Division of Neuroscience and Clinical Pharmacology, University of Cagliari, 09042 Monserrato, Italy; saghedduclaudia@gmail.com (C.S.); mpistis@unica.it (M.P.)

8 Section of Cagliari, Neuroscience Institute, National Research Council of Italy (CNR), 09042 Cagliari, Italy

9 Department of Psychological Sciences, Behavioral Neuroscience Division, University of Connecticut, 06269 Storrs, Connecticut, USA

10 Core Unit of Biomedical Research, Division of Laboratory Animal Science and Genetics, Medical University of Vienna, 2325 Himberg, Austria; harald.hoeger@meduniwien.ac.at

Functional GPCRs agonist and antagonist screening

This study was outsourced to Eurofins Cerep (Celle l'Evescault, France) and compliant with the corresponding project FR095-0004218.

Table S1 Agonist effect of S-CE-123 at 2.8E-06 M (IC50 for DAT re-uptake inhibition) in functional cellular assay. The results are expressed as a percent of control agonist response: (measured response / control response) x 100 obtained in the presence of S-CE-123.

Compound I.D.	Client Compound I.D.	Test Concentration	% of Control Agonist Response		
			1st	2nd	Mean
**A1 *(h)* (agonist effect)**					
100043705-1	S-CE-123	2.8E-06 M	5.3	0.8	3.0
**A_{2A} (agonist effect)**					
100043705-1	S-CE-123	2.8E-06 M	-0.9	1.2	0.2
**A_{2B} *(h)* (agonist effect)**					
100043705-1	S-CE-123	2.8E-06 M	0.5	3.4	1.9
**A_3 *(h)* (agonist effect)**					
100043705-1	S-CE-123	2.8E-06 M	-7.5	-41.0	-24.3
**α_{1A} *(h)* (agonist effect)**					
100043705-1	S-CE-123	2.8E-06 M	-2.5	-2.4	-2.5
**α_{1B} *(h)* (agonist effect)**					
100043705-1	S-CE-123	2.8E-06 M	7.2	-8.5	-0.6
**alpha $_{1D}$ *(h)* (agonist effect)**					
100043705-1	S-CE-123	2.8E-06 M	0.6	0.6	0.6
**α2A *(h)* (agonist effect)**					
100043705-1	S-CE-123	2.8E-06 M	2.3	1.7	2.0
**α_{2B} *(h)* (agonist effect)**					
100043705-1	S-CE-123	2.8E-06 M	-3.6	-4.9	-4.2
**α_{2C} *(h)* (agonist effect)**					
100043705-1	S-CE-123	2.8E-06 M	4.0	6.2	5.1
**β_1 *(h)* (agonist effect)**					
100043705-1	S-CE-123	2.8E-06 M	-0.1	-3.9	-2.0
**β_2 *(h)* (agonist effect)**					
100043705-1	S-CE-123	2.8E-06 M	-2.1	1.0	-0.6
**β_3 *(h)* (agonist effect)**					
100043705-1	S-CE-123	2.8E-06 M	-3.2	3.8	0.3
**AT_1 *(h)* (agonist effect)**					
100043705-1	S-CE-123	2.8E-06 M	-3.6	-1.2	-2.4
**APJ (apelin) *(h)* (agonist effect)**					
100043705-1	S-CE-123	2.8E-06 M	0.0	-13.1	-6.5
**TGR5 (h) (agonist effect)**					
100043705-1	S-CE-123	2.8E-06 M	3.3	1.4	2.3
**BB_1 *(h)* (agonist effect)**					
100043705-1	S-CE-123	2.8E-06 M	-1.1	-3.7	-2.4
**BB_2 *(h)* (agonist effect)**					
100043705-1	S-CE-123	2.8E-06 M	-0.3	0.8	0.3
**BB_3 *(h)* (agonist effect)**					
100043705-1	S-CE-123	2.8E-06 M	-6.8	-5.9	-6.3
**B_1 *(h)* (agonist effect)**					
100043705-1	S-CE-123	2.8E-06 M	-12.4	-11.5	-11.9
**B_2 *(h)* (agonist effect)**					
100043705-1	S-CE-123	2.8E-06 M	-5.6	-1.0	-3.3
**CGRP *(h)* (agonist effect)**					
100043705-1	S-CE-123	2.8E-06 M	7.4	7.2	7.3
**CT (Calcitonin) *(h)* (agonist effect)**					
100043705-1	S-CE-123	2.8E-06 M	3.4	0.9	2.2
**CaS *(h)* (agonist effect)**					
100043705-1	S-CE-123	2.8E-06 M	2.2	1.9	2.0
**CB_1 *(h)* (agonist effect)**					
100043705-1	S-CE-123	2.8E-06 M	-1.3	5.2	2.0
**CB_2 *(h)* (agonist effect)**					
100043705-1	S-CE-123	2.8E-06 M	-16.9	25.9	4.5
**CCR1 *(h)* (agonist effect)**					
100043705-1	S-CE-123	2.8E-06 M	8.1	6.8	7.4
**CCR2 *(h)* (agonist effect)**					
100043705-1	S-CE-123	2.8E-06 M	5.5	2.7	4.1

Compound I.D.	Client Compound I.D.	Test Concentration	% of Control Agonist Response 1st	2nd	Mean
**CCR3 *(h)* (agonist effect)**					
100043705-1	S-CE-123	2.8E-06 M	1.4	1.2	1.3
**CCR4 *(h)* (agonist effect)**					
100043705-1	S-CE-123	2.8E-06 M	-0.5	-0.3	-0.4
**CCR5 (agonist effect)**					
100043705-1	S-CE-123	2.8E-06 M	-0.8	-0.2	-0.5
**CCR6 *(h)* (agonist effect)**					
100043705-1	S-CE-123	2.8E-06 M	-1.8	-2.1	-1.9
**CCR7 *(h)* (agonist effect)**					
100043705-1	S-CE-123	2.8E-06 M	1.2	0.2	0.7
**CCR8 *(h)* (agonist effect)**					
100043705-1	S-CE-123	2.8E-06 M	3.1	3.8	3.4
**CCR10 *(h)* (agonist effect)**					
100043705-1	S-CE-123	2.8E-06 M	0.0	0.3	0.2
**CX3CR1 *(h)* (agonist effect)**					
100043705-1	S-CE-123	2.8E-06 M	3.2	1.7	2.4
**CXCR1 *(h)* (agonist effect)**					
100043705-1	S-CE-123	2.8E-06 M	0.0	0.3	0.1
**CXCR2 *(h)* (agonist effect)**					
100043705-1	S-CE-123	2.8E-06 M	1.9	-2.9	-0.5
**CXCR3 (h) (agonist effect)**					
100043705-1	S-CE-123	2.8E-06 M	1.1	-0.4	0.4
**CXCR4 *(h)* (agonist effect)**					
100043705-1	S-CE-123	2.8E-06 M	-0.3	-0.2	-0.2
**CXCR5 *(h)* (agonist effect)**					
100043705-1	S-CE-123	2.8E-06 M	1.0	0.9	0.9
**CXCR6 *(h)* (agonist effect)**					
100043705-1	S-CE-123	2.8E-06 M	-0.4	-1.2	-0.8
**CCK_1 (CCK_A) *(h)* (agonist effect)**					
100043705-1	S-CE-123	2.8E-06 M	0.4	0.6	0.5
**CCK_2 (CCK_B) *(h)* (agonist effect)**					
100043705-1	S-CE-123	2.8E-06 M	-2.2	0.2	-1.0
**C3aR *(h)* (agonist effect)**					
100043705-1	S-CE-123	2.8E-06 M	-0.5	-0.1	-0.3
**ChemR23 *(h)* (agonist effect)**					
100043705-1	S-CE-123	2.8E-06 M	0.0	1.4	0.7
**CRF_1 *(h)* (agonist effect)**					
100043705-1	S-CE-123	2.8E-06 M	-1.2	-3.7	-2.5
**$CRF_{2\alpha}$ *(h)* (agonist effect)**					
100043705-1	S-CE-123	2.8E-06 M	-1.4	-2.2	-1.8
**XCR1 / GPR5 *(h)* (agonist effect)**					
100043705-1	S-CE-123	2.8E-06 M	2.3	0.4	1.3
**D_1 *(h)* (agonist effect)**					
100043705-1	S-CE-123	2.8E-06 M	0.2	-0.5	-0.1
**D_{2S} *(h)* (agonist effect)**					
100043705-1	S-CE-123	2.8E-06 M	9.2	11.7	10.5
**D_3 *(h)* (agonist effect)**					
100043705-1	S-CE-123	2.8E-06 M	-20.1	5.7	-7.2
**$D_{4.4}$ *(h)* (agonist effect)**					
100043705-1	S-CE-123	2.8E-06 M	-10.2	-1.6	-5.9
**D_5 *(h)* (agonist effect)**					
100043705-1	S-CE-123	2.8E-06 M	-2.0	0.6	-0.7
**ET_A *(h)* (agonist effect)**					
100043705-1	S-CE-123	2.8E-06 M	1.0	1.4	1.2
**ET_B *(h)* (agonist effect)**					
100043705-1	S-CE-123	2.8E-06 M	-0.5	2.7	1.1

Compound I.D.	Client Compound I.D.	Test Concentration	% of Control Agonist Response 1st	2nd	Mean
**FFA1 *(h)* (GPR40) (agonist effect)**					
100043705-1	S-CE-123	2.8E-06 M	-2.6	-2.7	-2.7
**FFA2 *(h)* (GPR43) (agonist effect)**					
100043705-1	S-CE-123	2.8E-06 M	6.4	6.7	6.6
**FFA3 *(h)* (GPR41) (agonist effect)**					
100043705-1	S-CE-123	2.8E-06 M	-19.5	11.7	-3.9
**FFA4 (GPR120) *(h)* (agonist effect)**					
100043705-1	S-CE-123	2.8E-06 M	0.3	-0.5	-0.1
**GnRH *(h)* (agonist effect)**					
100043705-1	S-CE-123	2.8E-06 M	-0.6	-0.7	-0.6
**GPR39 *(h)* (agonist effect)**					
100043705-1	S-CE-123	2.8E-06 M	-0.8	1.9	0.6
**OXGR1 GPR99 *(h)* (agonist effect)**					
100043705-1	S-CE-123	2.8E-06 M	1.1	0.6	0.9
**GPR103/QRFP *(h)* (agonist effect)**					
100043705-1	S-CE-123	2.8E-06 M	0.5	-0.3	0.1
**GPR109A *(h)* (agonist effect)**					
100043705-1	S-CE-123	2.8E-06 M	0.5	0.4	0.4
**GPR119 (agonist effect)**					
100043705-1	S-CE-123	2.8E-06 M	11.5	-9.6	0.9
**FPR1 *(h)* (agonist effect)**					
100043705-1	S-CE-123	2.8E-06 M	-46.5	-6.4	-26.5
**GABAB1b beta *(h)* (agonist effect)**					
100043705-1	S-CE-123	2.8E-06 M	-0.3	0.2	0.0
**GAL1 *(h)* (agonist effect)**					
100043705-1	S-CE-123	2.8E-06 M	-0.8	-1.7	-1.3
**GAL_2 *(h)* (agonist effect)**					
100043705-1	S-CE-123	2.8E-06 M	5.2	3.4	4.3
**GIP *(h)* (agonist effect)**					
100043705-1	S-CE-123	2.8E-06 M	-0.1	0.3	0.1
**GLP-1 (agonist effect)**					
100043705-1	S-CE-123	2.8E-06 M	10.7	13.4	12.0
**secretin *(h)* (agonist effect)**					
100043705-1	S-CE-123	2.8E-06 M	-1.1	2.7	0.8
**TSH *(h)* (agonist effect)**					
100043705-1	S-CE-123	2.8E-06 M	-4.3	-3.6	-3.9
**GHRH *(h)* (agonist effect)**					
100043705-1	S-CE-123	2.8E-06 M	0.2	2.4	1.3
**Ghrelin / GHSR-1a *(h)* (agonist effect)**					
100043705-1	S-CE-123	2.8E-06 M	1.7	2.6	2.2
**H_1 *(h)* (agonist effect)**					
100043705-1	S-CE-123	2.8E-06 M	-1.7	-0.7	-1.2
**H_2 *(h)* (agonist effect)**					
100043705-1	S-CE-123	2.8E-06 M	-4.4	-0.6	-2.5
**H_3 *(h)* (agonist effect)**					
100043705-1	S-CE-123	2.8E-06 M	-2.6	1.0	-0.8
**H_4 *(h)* (agonist effect)**					
100043705-1	S-CE-123	2.8E-06 M	-0.2	1.7	0.7
**KiSS1 / GPR54 *(h)* (agonist effect)**					
100043705-1	S-CE-123	2.8E-06 M	-0.3	-0.7	-0.5
**BLT_1 (LTB_4) *(h)* (agonist effect)**					
100043705-1	S-CE-123	2.8E-06 M	-0.6	-1.4	-1.0
**$CysLT_1$ (LTD_4) *(h)* (agonist effect)**					
100043705-1	S-CE-123	2.8E-06 M	-9.7	-6.8	-8.2
**$CysLT_2$ (LTC_4) *(h)* (agonist effect)**					
100043705-1	S-CE-123	2.8E-06 M	-0.5	-1.9	-1.2

Compound I.D.	Client Compound I.D.	Test Concentration	% of Control Agonist Response 1st	2nd	Mean
**LPA1(h) (agonist effect)**					
100043705-1	S-CE-123	2.8E-06 M	-0.1	-0.2	-0.2
**LPA_2 *(h)* (agonist effect)**					
100043705-1	S-CE-123	2.8E-06 M	1.7	4.6	3.2
**LPA_3 *(h)* (agonist effect)**					
100043705-1	S-CE-123	2.8E-06 M	-11.6	2.6	-4.5
**S_1P_1 *(h)* (agonist effect)**					
100043705-1	S-CE-123	2.8E-06 M	8.3	11.5	9.9
**S_1P_2 *(h)* (agonist effect)**					
100043705-1	S-CE-123	2.8E-06 M	0.7	-8.9	-4.1
**S_1P_3 *(h)* (agonist effect)**					
100043705-1	S-CE-123	2.8E-06 M	-1.3	-1.1	-1.2
**S1P4 *(h)* (agonist effect)**					
100043705-1	S-CE-123	2.8E-06 M	-4.2	-8.3	-6.2
**S1P5 *(h)* (agonist effect)**					
100043705-1	S-CE-123	2.8E-06 M	-0.1	-0.4	-0.2
**MCH_1 *(h)* (agonist effect)**					
100043705-1	S-CE-123	2.8E-06 M	-0.8	1.0	0.1
**MCH_2 *(h)* (agonist effect)**					
100043705-1	S-CE-123	2.8E-06 M	3.6	5.7	4.6
**MC_1 (agonist effect)**					
100043705-1	S-CE-123	2.8E-06 M	-2.0	-2.1	-2.1
**MC_2 *(h)* (agonist effect)**					
100043705-1	S-CE-123	2.8E-06 M	-3.8	-1.6	-2.7
**MC_3 *(h)* (agonist effect)**					
100043705-1	S-CE-123	2.8E-06 M	-2.5	0.3	-1.1
**MC_4 *(h)* (agonist effect)**					
100043705-1	S-CE-123	2.8E-06 M	-3.2	-0.4	-1.8
**MC_5 *(h)* (agonist effect)**					
100043705-1	S-CE-123	2.8E-06 M	3.0	3.0	3.0
**MT_1 (ML_{1A}) *(h)* (agonist effect)**					
100043705-1	S-CE-123	2.8E-06 M	15.2	18.4	16.8
**MT_2 (ML_{1B}) *(h)* (agonist effect)**					
100043705-1	S-CE-123	2.8E-06 M	-0.8	-59.1	-29.9
**motilin *(h)* (agonist effect)**					
100043705-1	S-CE-123	2.8E-06 M	-0.7	-2.7	-1.7
**M_1 *(h)* (agonist effect)**					
100043705-1	S-CE-123	2.8E-06 M	-1.1	-0.7	-0.9
**M_2 *(h)* (agonist effect)**					
100043705-1	S-CE-123	2.8E-06 M	16.8	17.3	17.1
**M_3 *(h)* (agonist effect)**					
100043705-1	S-CE-123	2.8E-06 M	0.0	0.3	0.1
**M_4 *(h)* (agonist effect)**					
100043705-1	S-CE-123	2.8E-06 M	1.7	1.9	1.8
**M_5 *(h)* (agonist effect)**					
100043705-1	S-CE-123	2.8E-06 M	0.0	-0.2	-0.1
**MrgD *(h)* (agonist effect)**					
100043705-1	S-CE-123	2.8E-06 M	-2.0	3.5	0.7
**MRGX1 / MRGPRX1 *(h)* (agonist effect)**					
100043705-1	S-CE-123	2.8E-06 M	-10.7	0.2	-5.2
**MRGX2 / MGRPLX2 *(h)* (agonist effect)**					
100043705-1	S-CE-123	2.8E-06 M	1.7	2.6	2.2
**NPS *(h)* (agonist effect)**					
100043705-1	S-CE-123	2.8E-06 M	-3.3	1.3	-1.0
**NPBW1 *(h)* (agonist effect)**					
100043705-1	S-CE-123	2.8E-06 M	-3.1	15.5	6.2

Compound I.D.	Client Compound I.D.	Test Concentration	% of Control Agonist Response 1st	2nd	Mean
**NK_1 *(h)* (agonist effect)**					
100043705-1	S-CE-123	2.8E-06 M	-0.1	-0.1	-0.1
**NK_2 *(h)* (agonist effect)**					
100043705-1	S-CE-123	2.8E-06 M	-0.3	-1.4	-0.9
**NK_3 *(h)* (agonist effect)**					
100043705-1	S-CE-123	2.8E-06 M	0.5	-0.8	-0.2
**NMU1 *(h)* (agonist effect)**					
100043705-1	S-CE-123	2.8E-06 M	-0.7	-1.2	-0.9
**NMU2 *(h)* (agonist effect)**					
100043705-1	S-CE-123	2.8E-06 M	0.1	0.0	0.0
**NTS_1 (NT_1) *(h)* (agonist effect)**					
100043705-1	S-CE-123	2.8E-06 M	1.6	-2.8	-0.6
**NTS_2 (NT2) *(h)* (agonist effect)**					
100043705-1	S-CE-123	2.8E-06 M	0.8	2.9	1.9
**δ (DOP) *(h)* (agonist effect)**					
100043705-1	S-CE-123	2.8E-06 M	1.3	0.9	1.1
**κ (KOP) (agonist effect)**					
100043705-1	S-CE-123	2.8E-06 M	33.6	9.3	21.4
**μ (MOP) *(h)* (agonist effect)**					
100043705-1	S-CE-123	2.8E-06 M	1.6	-4.4	-1.4
**NOP *(h)* (ORL1) (agonist effect)**					
100043705-1	S-CE-123	2.8E-06 M	20.7	-3.6	8.6
**OX_1 *(h)* (agonist effect)**					
100043705-1	S-CE-123	2.8E-06 M	-9.5	-8.8	-9.2
**OX_2 *(h)* (agonist effect)**					
100043705-1	S-CE-123	2.8E-06 M	1.5	0.3	0.9
**PAF *(h)* (agonist effect)**					
100043705-1	S-CE-123	2.8E-06 M	1.8	2.0	1.9
**PTH1 *(h)* (agonist effect)**					
100043705-1	S-CE-123	2.8E-06 M	-3.8	-2.9	-3.4
**PK_1 *(h)* (agonist effect)**					
100043705-1	S-CE-123	2.8E-06 M	-5.1	0.6	-2.3
**PK_2 *(h)* (agonist effect)**					
100043705-1	S-CE-123	2.8E-06 M	-0.8	-2.4	-1.6
**DP_1 *(h)* (agonist effect)**					
100043705-1	S-CE-123	2.8E-06 M	-1.0	-4.3	-2.7
**EP_1 *(h)* (agonist effect)**					
100043705-1	S-CE-123	2.8E-06 M	4.6	0.8	2.7
**EP_2 *(h)* (agonist effect)**					
100043705-1	S-CE-123	2.8E-06 M	1.8	2.2	2.0
**EP3 *(h)* (agonist effect)**					
100043705-1	S-CE-123	2.8E-06 M	2.3	2.2	2.3
**EP_4 *(h)* (agonist effect)**					
100043705-1	S-CE-123	2.8E-06 M	4.6	-0.3	2.2
**FP *(h)* (agonist effect)**					
100043705-1	S-CE-123	2.8E-06 M	-1.9	-1.9	-1.9
**IP (PGI_2) *(h)* (agonist effect)**					
100043705-1	S-CE-123	2.8E-06 M	0.6	-1.6	-0.5
**TP (TXA_2/PGH_2) *(h)* (agonist effect)**					
100043705-1	S-CE-123	2.8E-06 M	-0.4	-1.0	-0.7
**PAR1 *(h)* (agonist effect)**					
100043705-1	S-CE-123	2.8E-06 M	0.6	0.0	0.3
**PAR2 *(h)* (agonist effect)**					
100043705-1	S-CE-123	2.8E-06 M	-0.9	15.0	7.1
**P2Y1 *(h)* (agonist effect)**					
100043705-1	S-CE-123	2.8E-06 M	0.9	3.1	2.0

Compound I.D.	Client Compound I.D.	Test Concentration	% of Control Agonist Response		
			1st	2nd	Mean
**$P2Y_2$ *(h)* (agonist effect)**					
100043705-1	S-CE-123	2.8E-06 M	2.4	-1.1	0.6
**$P2Y_4$ *(h)* (agonist effect)**					
100043705-1	S-CE-123	2.8E-06 M	-0.8	-1.2	-1.0
**$P2Y_6$ *(h)* (agonist effect)**					
100043705-1	S-CE-123	2.8E-06 M	-4.0	0.3	-1.8
**P2Y11 *(h)* (agonist effect)**					
100043705-1	S-CE-123	2.8E-06 M	0.3	3.0	1.7
**PTH2 *(h)* (agonist effect)**					
100043705-1	S-CE-123	2.8E-06 M	-4.1	-4.5	-4.3
**RXFP1 *(h)* (agonist effect)**					
100043705-1	S-CE-123	2.8E-06 M	-3.5	-0.5	-2.0
**5-HT1A *(h)* (agonist effect)**					
100043705-1	S-CE-123	2.8E-06 M	0.6	-0.6	0.0
**5HT1B (h) (agonist effect)**					
100043705-1	S-CE-123	2.8E-06 M	-30.4	-31.0	-30.7
**$5\text{-}HT_{1D}$ (agonist effect)**					
100043705-1	S-CE-123	2.8E-06 M	3.4	2.1	2.8
**$5\text{-}HT_{2A}$ *(h)* (agonist effect)**					
100043705-1	S-CE-123	2.8E-06 M	2.0	-2.5	-0.3
**$5\text{-}HT_{2B}$ *(h)* (agonist effect)**					
100043705-1	S-CE-123	2.8E-06 M	0.9	4.4	2.7
**$5\text{-}HT_{2C}$ *(h)* (agonist effect)**					
100043705-1	S-CE-123	2.8E-06 M	1.4	-0.8	0.3
**$5\text{-}HT_{4e}$ *(h)* (agonist effect)**					
100043705-1	S-CE-123	2.8E-06 M	-2.9	0.0	-1.4
**$5\text{-}HT_6$ *(h)* (agonist effect)**					
100043705-1	S-CE-123	2.8E-06 M	1.9	-5.5	-1.8
**$5\text{-}HT_7$ *(h)* (agonist effect)**					
100043705-1	S-CE-123	2.8E-06 M	-3.5	3.2	-0.2
**sst_1 *(h)* (agonist effect)**					
100043705-1	S-CE-123	2.8E-06 M	-20.0	-1.7	-10.9
**sst2 *(h)* (agonist effect)**					
100043705-1	S-CE-123	2.8E-06 M	0.6	-0.5	0.1
**sst3 *(h)* (agonist effect)**					
100043705-1	S-CE-123	2.8E-06 M	0.9	0.4	0.6
**sst_4 *(h)* (agonist effect)**					
100043705-1	S-CE-123	2.8E-06 M	24.2	19.9	22.0
**sst_5 *(h)* (agonist effect)**					
100043705-1	S-CE-123	2.8E-06 M	6.4	45.7	26.0
**SUCNR1/GPR91 *(h)* (agonist effect)**					
100043705-1	S-CE-123	2.8E-06 M	-0.2	-0.2	-0.2
**TRH_1 *(h)* (agonist effect)**					
100043705-1	S-CE-123	2.8E-06 M	-1.0	-0.6	-0.8
**UT *(h)* (agonist effect)**					
100043705-1	S-CE-123	2.8E-06 M	0.5	1.3	0.9
**PAC_1 (PACAP) *(h)* (agonist effect)**					
100043705-1	S-CE-123	2.8E-06 M	-0.1	0.1	0.0
**$VPAC_1$ (VIP_1) *(h)* (agonist effect)**					
100043705-1	S-CE-123	2.8E-06 M	-2.5	-1.2	-1.8
**$VPAC_2$ (VIP_2) *(h)* (agonist effect)**					
100043705-1	S-CE-123	2.8E-06 M	-5.7	0.8	-2.5
**Y4 *(h)* (agonist effect)**					
100043705-1	S-CE-123	2.8E-06 M	0.2	0.1	0.1
**V_{1a} *(h)* (agonist effect)**					
100043705-1	S-CE-123	2.8E-06 M	-2.8	-1.5	-2.1
**V1B *(h)* (agonist effect)**					
100043705-1	S-CE-123	2.8E-06 M	0.9	-0.3	0.3
**V_2 *(h)* (agonist effect)**					
100043705-1	S-CE-123	2.8E-06 M	-0.4	-6.1	-3.3

Table S2 Reference compounds for agonist effect.

Compound I.D.	EC_{50} (M)	nH
**A1 *(h)* (agonist effect)**		
CPA	1.6E-09 M	n/a
**A_{2A} (agonist effect)**		
NECA	1.3E-08 M	n/a
**A_{2B} *(h)* (agonist effect)**		
NECA	8.5E-08 M	n/a
**A_3 *(h)* (agonist effect)**		
IB-MECA	5.6E-10 M	n/a
**α_{1A} *(h)* (agonist effect)**		
epinephrine	5.8E-10 M	n/a
**α_{1B} *(h)* (agonist effect)**		
epinephrine	1.9E-07 M	n/a
**alpha $_{1D}$ *(h)* (agonist effect)**		
Epinephrine	1.6E-09 M	n/a
**α2A *(h)* (agonist effect)**		
Epinephrine	3.3E-10 M	n/a
**α_{2B} *(h)* (agonist effect)**		
dexmedetomidine	1.9E-08 M	n/a
**α_{2C} *(h)* (agonist effect)**		
epinephrine	1.4E-09 M	n/a
**β_1 *(h)* (agonist effect)**		
isoproterenol	6.0E-10 M	n/a
**β_2 *(h)* (agonist effect)**		
isoproterenol	3.9E-09 M	n/a
**β_3 *(h)* (agonist effect)**		
isoproterenol	4.7E-07 M	n/a
**AT_1 *(h)* (agonist effect)**		
angiotensin-II	1.2E-10 M	n/a
**APJ (apelin) *(h)* (agonist effect)**		
apelin-13	7.3E-11 M	n/a
**TGR5 (h) (agonist effect)**		
Lithocholic Acid	8.2E-07 M	n/a
**BB_1 *(h)* (agonist effect)**		
neuromedin B	5.0E-13 M	n/a
**BB_2 *(h)* (agonist effect)**		
GRP	2.7E-11 M	n/a
**BB_3 *(h)* (agonist effect)**		
Bn(6-14)	2.1E-09 M	n/a
**B_1 *(h)* (agonist effect)**		
LysdesArg^9-BK	2.1E-10 M	n/a
**B_2 *(h)* (agonist effect)**		
bradykinin	5.1E-12 M	n/a
**CGRP *(h)* (agonist effect)**		
hCGRPα	4.5E-11 M	n/a
**CT (Calcitonin) *(h)* (agonist effect)**		
human calcitonin	8.6E-10 M	n/a
**CaS *(h)* (agonist effect)**		
neomycin	1.7E-05 M	n/a

Compound I.D.	EC_{50} (M)	nH
**CB_1 *(h)* (agonist effect)**		
CP 55940	5.1E-11 M	n/a
**CB_2 *(h)* (agonist effect)**		
WIN 55212-2	1.4E-10 M	n/a
**CCR1 *(h)* (agonist effect)**		
MIP-1alpha	1.2E-10 M	n/a
**CCR2 *(h)* (agonist effect)**		
MCP-1	1.5E-09 M	n/a
**CCR3 *(h)* (agonist effect)**		
Human Eotaxin(CCL11)	1.7E-09 M	n/a
**CCR4 *(h)* (agonist effect)**		
TARC	2.4E-10 M	n/a
**CCR5 (agonist effect)**		
MIP-1a	5.0E-09 M	n/a
**CCR6 *(h)* (agonist effect)**		
MIP-3a	1.8E-10 M	n/a
**CCR7 *(h)* (agonist effect)**		
MIP-3b	3.4E-10 M	n/a
**CCR8 *(h)* (agonist effect)**		
I-309	2.5E-10 M	n/a
**CCR10 *(h)* (agonist effect)**		
CTACK/CCL27	6.5E-09 M	n/a
**CX3CR1 *(h)* (agonist effect)**		
Human Fractalkine(CX3CL1)	9.0E-10 M	n/a
**CXCR1 *(h)* (agonist effect)**		
rhIL-8	2.9E-10 M	n/a
**CXCR2 *(h)* (agonist effect)**		
rhGROa	4.9E-10 M	n/a
**CXCR3 (h) (agonist effect)**		
I-TAC	3.2E-09 M	n/a
**CXCR4 *(h)* (agonist effect)**		
SDF-1a	2.4E-10 M	n/a
**CXCR5 *(h)* (agonist effect)**		
CXCL13/BLC/BCA-1	2.5E-09 M	n/a
**CXCR6 *(h)* (agonist effect)**		
CXCL16	1.7E-09 M	n/a
**CCK_1 (CCK_A) *(h)* (agonist effect)**		
CCK-8s	6.3E-08 M	n/a
**CCK_2 (CCK_B) *(h)* (agonist effect)**		
CCK-8s	2.4E-09 M	n/a
**C3aR *(h)* (agonist effect)**		
Complement C3a human	7.0E-10 M	n/a
**ChemR23 *(h)* (agonist effect)**		
Chemerin	9.6E-10 M	n/a
**CRF_1 *(h)* (agonist effect)**		
ovine CRF	9.6E-09 M	n/a
**$CRF_{2\alpha}$ *(h)* (agonist effect)**		
human CRF	4.7E-08 M	n/a
**XCR1 / GPR5 *(h)* (agonist effect)**		
Lymphotactin	1.3E-09 M	n/a
**D_1 *(h)* (agonist effect)**		
dopamine	2.1E-08 M	n/a
**D_{2S} *(h)* (agonist effect)**		
dopamine	3.3E-09 M	n/a
**D_3 *(h)* (agonist effect)**		
dopamine	3.5E-09 M	n/a
**$D_{4.4}$ *(h)* (agonist effect)**		
dopamine	1.2E-08 M	n/a

Compound I.D.	EC_{50} (M)	nH
**D_5 *(h)* (agonist effect)**		
dopamine	1.1E-08 M	n/a
**ET_A *(h)* (agonist effect)**		
endothelin-1	4.5E-10 M	n/a
**ET_B *(h)* (agonist effect)**		
endothelin-1	1.7E-10 M	n/a
**FFA1 *(h)* (GPR40) (agonist effect)**		
linoleic acid	6.2E-06 M	n/a
**FFA2 *(h)* (GPR43) (agonist effect)**		
sodium acetate	1.9E-04 M	n/a
**FFA3 *(h)* (GPR41) (agonist effect)**		
sodium propionate	2.0E-06 M	n/a
**FFA4 (GPR120) *(h)* (agonist effect)**		
GW9508	7.4E-06 M	n/a
**GnRH *(h)* (agonist effect)**		
LHRH	4.1E-10 M	n/a
**GPR39 *(h)* (agonist effect)**		
ZnCl2	1.7E-05 M	n/a
**OXGR1 GPR99 *(h)* (agonist effect)**		
alpha Ketoglutaric acid	1.8E-04 M	n/a
**GPR103/QRFP *(h)* (agonist effect)**		
26Rfa Hypothalamic Peptide(Peptide 518)	4.9E-10 M	n/a
**GPR109A *(h)* (agonist effect)**		
Nicotinic Acid Free Acid	4.7E-08 M	n/a
**GPR119 (agonist effect)**		
AR231453	3.4E-08 M	n/a
**FPR1 *(h)* (agonist effect)**		
fMLP	1.6E-10 M	n/a
**GABAB1b beta *(h)* (agonist effect)**		
3-APMPA	1.2E-07 M	n/a
**GAL1 *(h)* (agonist effect)**		
Galanin (1-30)	1.4E-10 M	n/a
**GAL_2 *(h)* (agonist effect)**		
human galanin	3.4E-10 M	n/a
**GIP *(h)* (agonist effect)**		
GIP	6.1E-10 M	n/a
**GLP-1 (agonist effect)**		
GLP-1(7-37)	7.6E-11 M	n/a
**secretin *(h)* (agonist effect)**		
human secretin	1.2E-10 M	n/a
**TSH *(h)* (agonist effect)**		
TSH	2.2E-08 M	n/a
**GHRH *(h)* (agonist effect)**		
human GHRF(1-29)	2.3E-09 M	n/a
**Ghrelin / GHSR-1a *(h)* (agonist effect)**		
Ghrelin(human)	4.0E-10 M	n/a
**H_1 *(h)* (agonist effect)**		
histamine	4.7E-08 M	n/a
**H_2 *(h)* (agonist effect)**		
histamine	4.8E-07 M	n/a
**H_3 *(h)* (agonist effect)**		
histamine	3.2E-08 M	n/a
**H_4 *(h)* (agonist effect)**		
histamine	4.8E-09 M	n/a
**KiSS1 / GPR54 *(h)* (agonist effect)**		
Metastin 45-54	2.4E-09 M	n/a
**BLT_1 (LTB_4) *(h)* (agonist effect)**		
LTB_4	4.6E-10 M	n/a

Compound I.D.	EC_{50} (M)	nH
**NPBW1 *(h)* (agonist effect)**		
NPB29	1.8E-10 M	n/a
**NK_1 *(h)* (agonist effect)**		
$[Sar^9,Met(O_2)^{11}]$-SP	6.3E-11 M	n/a
**NK_2 *(h)* (agonist effect)**		
$[Nleu^{10}]$-NKA (4-10)	1.3E-09 M	n/a
**NK_3 *(h)* (agonist effect)**		
$[MePhe^7]$-NKB	4.9E-11 M	n/a
**NMU1 *(h)* (agonist effect)**		
NMU-25 (human)	1.0E-10 M	n/a
**NMU2 *(h)* (agonist effect)**		
Neuromedian U-25	6.3E-10 M	n/a
**NTS_1 (NT_1) *(h)* (agonist effect)**		
neurotensin	1.5E-11 M	n/a
**NTS_2 (NT2) *(h)* (agonist effect)**		
SR 142948	3.3E-09 M	n/a
**δ (DOP) *(h)* (agonist effect)**		
DPDPE	3.6E-09 M	n/a
**κ (KOP) (agonist effect)**		
U 50488	2.8E-10 M	n/a
**μ (MOP) *(h)* (agonist effect)**		
DAMGO	1.8E-09 M	n/a
**NOP *(h)* (ORL1) (agonist effect)**		
nociceptin	3.1E-11 M	n/a
**OX_1 *(h)* (agonist effect)**		
orexin-A	3.4E-10 M	n/a
**OX_2 *(h)* (agonist effect)**		
orexin-B	9.5E-10 M	n/a
**PAF *(h)* (agonist effect)**		
PAF(C16)	4.9E-10 M	n/a
**PTH1 *(h)* (agonist effect)**		
PTHrP (1-34)	1.0E-10 M	n/a
**PK_1 *(h)* (agonist effect)**		
PK1	1.7E-09 M	n/a
**PK_2 *(h)* (agonist effect)**		
PK2	5.3E-10 M	n/a
**DP_1 *(h)* (agonist effect)**		
BW 245C	2.2E-09 M	n/a
**EP_1 *(h)* (agonist effect)**		
PGE_2	1.6E-10 M	n/a
**EP_2 *(h)* (agonist effect)**		
PGE_2	1.2E-08 M	n/a
**EP3 *(h)* (agonist effect)**		
Prostaglandin E2	1.2E-09 M	n/a
**EP_4 *(h)* (agonist effect)**		
PGE_2	3.2E-09 M	n/a
**FP *(h)* (agonist effect)**		
PGF2alpha	5.2E-10 M	n/a
**IP (PGI_2) *(h)* (agonist effect)**		
iloprost	3.7E-10 M	n/a
**TP (TXA_2/PGH_2) *(h)* (agonist effect)**		
U 44069	1.3E-09 M	n/a
**PAR1 *(h)* (agonist effect)**		
TFLLR-NH_2	1.4E-06 M	n/a
**PAR2 *(h)* (agonist effect)**		
trypsin	1.2E-08 M	n/a
**P2Y1 *(h)* (agonist effect)**		
2MeSATP	6.0E-10 M	n/a

Compound I.D.	EC_{50} (M)	nH
**$CysLT_1$ (LTD_4) *(h)* (agonist effect)**		
LTD_4	2.6E-11 M	n/a
**$CysLT_2$ (LTC_4) *(h)* (agonist effect)**		
LTC_4	1.7E-09 M	n/a
**LPA1(h) (agonist effect)**		
Oleoyl-LPA	1.0E-07 M	n/a
**LPA_2 *(h)* (agonist effect)**		
LPA	3.8E-09 M	n/a
**LPA_3 *(h)* (agonist effect)**		
LPA	9.2E-09 M	n/a
**S_1P_1 *(h)* (agonist effect)**		
S1P	8.1E-10 M	n/a
**S_1P_2 *(h)* (agonist effect)**		
S_1P	1.1E-08 M	n/a
**S_1P_3 *(h)* (agonist effect)**		
S_1P	1.1E-09 M	n/a
**S1P4 *(h)* (agonist effect)**		
Sphingosine 1-phosphate (S1P)	5.2E-09 M	n/a
**S1P5 *(h)* (agonist effect)**		
Sphingosine 1-phosphate (S1P)	1.3E-08 M	n/a
**MCH_1 *(h)* (agonist effect)**		
human MCH	1.4E-09 M	n/a
**MCH_2 *(h)* (agonist effect)**		
human MCH	8.4E-10 M	n/a
**MC_1 (agonist effect)**		
NDP-α-MSH	2.5E-10 M	n/a
**MC_2 *(h)* (agonist effect)**		
ACTH (1-39)	4.2E-09 M	n/a
**MC_3 *(h)* (agonist effect)**		
NDP-α-MSH	8.2E-10 M	n/a
**MC_4 *(h)* (agonist effect)**		
NDP-α-MSH	1.0E-10 M	n/a
**MC_5 *(h)* (agonist effect)**		
α-MSH	3.9E-07 M	n/a
**MT_1 (ML_{1A}) *(h)* (agonist effect)**		
melatonin	3.3E-11 M	n/a
**MT_2 (ML_{1B}) *(h)* (agonist effect)**		
melatonin	6.5E-11 M	n/a
**motilin *(h)* (agonist effect)**		
motilin	8.0E-12 M	n/a
**M_1 *(h)* (agonist effect)**		
acetylcholine	9.6E-10 M	n/a
**M_2 *(h)* (agonist effect)**		
acetylcholine	6.2E-08 M	n/a
**M_3 *(h)* (agonist effect)**		
acetylcholine	1.1E-08 M	n/a
**M_4 *(h)* (agonist effect)**		
acetylcholine	1.4E-08 M	n/a
**M_5 *(h)* (agonist effect)**		
Acetylcholine	1.1E-09 M	n/a
**MrgD *(h)* (agonist effect)**		
Beta-alanine	1.4E-06 M	n/a
**MRGX1 / MRGPRX1 *(h)* (agonist effect)**		
BAM(8-22)	2.1E-09 M	n/a
**MRGX2 / MGRPLX2 *(h)* (agonist effect)**		
Proadrenomedullin fragment, 1-20 (PAMP)	8.5E-08 M	n/a
**NPS *(h)* (agonist effect)**		
NPS	6.0E-10 M	n/a

Compound I.D.	EC_{50} (M)	nH
**$P2Y_2$ *(h)* (agonist effect)**		
UTP	4.4E-08 M	n/a
**$P2Y_4$ *(h)* (agonist effect)**		
UTP	5.6E-09 M	n/a
**$P2Y_6$ *(h)* (agonist effect)**		
UDP	1.1E-09 M	n/a
**P2Y11 *(h)* (agonist effect)**		
ATP	6.6E-06 M	n/a
**PTH2 *(h)* (agonist effect)**		
TIP-39	7.0E-09 M	n/a
**RXFP1 *(h)* (agonist effect)**		
H2 relaxin	5.5E-10 M	n/a
**5-HT1A *(h)* (agonist effect)**		
Serotonine	1.5E-09 M	n/a
**5HT1B (h) (agonist effect)**		
5-HT	2.1E-08 M	n/a
**$5\text{-}HT_{1D}$ (agonist effect)**		
serotonin	5.6E-10 M	n/a
**$5\text{-}HT_{2A}$ *(h)* (agonist effect)**		
serotonin	2.7E-08 M	n/a
**$5\text{-}HT_{2B}$ *(h)* (agonist effect)**		
serotonin	5.0E-09 M	n/a
**$5\text{-}HT_{2C}$ *(h)* (agonist effect)**		
serotonin	3.3E-09 M	n/a
**$5\text{-}HT_{4e}$ *(h)* (agonist effect)**		
serotonin	1.5E-09 M	n/a
**$5\text{-}HT_6$ *(h)* (agonist effect)**		
serotonin	6.0E-08 M	n/a
**$5\text{-}HT_7$ *(h)* (agonist effect)**		
serotonin	5.2E-08 M	n/a
**sst_1 *(h)* (agonist effect)**		
somatostatin-28	1.3E-10 M	n/a
**sst2 *(h)* (agonist effect)**		
Somatostatin	7.6E-10 M	n/a
**sst3 *(h)* (agonist effect)**		
Somatostatin	7.7E-10 M	n/a
**sst_4 *(h)* (agonist effect)**		
somatostatin-14	8.2E-11 M	n/a
**sst_5 *(h)* (agonist effect)**		
somatostatin-14	6.6E-09 M	n/a
**SUCNR1/GPR91 *(h)* (agonist effect)**		
Sodium Succinate	6.8E-05 M	n/a
**TRH_1 *(h)* (agonist effect)**		
TRH	7.2E-11 M	n/a
**UT *(h)* (agonist effect)**		
human urotensin-II	7.0E-10 M	n/a
**PAC_1 (PACAP) *(h)* (agonist effect)**		
$PACAP_{1\text{-}38}$	1.7E-10 M	n/a
**$VPAC_1$ (VIP_1) *(h)* (agonist effect)**		
VIP	1.6E-10 M	n/a
**$VPAC_2$ (VIP_2) *(h)* (agonist effect)**		
VIP	3.7E-09 M	n/a
**Y4 *(h)* (agonist effect)**		
Pancreatic polypeptide human	1.3E-10 M	n/a
**V_{1a} *(h)* (agonist effect)**		
AVP	2.3E-10 M	n/a
**V1B *(h)* (agonist effect)**		
Vasopressin	1.2E-10 M	n/a

Table S3 Antagonist effect of S-CE-123 at 2.8E-06 M (IC50 for DAT re-uptake inhibition) in functional cellular assay. The results are expressed as a percent inhibition of control agonist response: 100 –((measured response /control response) x 100).

Compound I.D.	Client Compound I.D.	Test Concentration	% Inhibition of Control Agonist Response		
			1st	2nd	Mean
**A1 *(h)* (antagonist effect)**					
100043705-1	S-CE-123	2.8E-06 M	6.3	14.9	10.6
**A_{2A} (antagonist effect)**					
100043705-1	S-CE-123	2.8E-06 M	20.0	-0.7	9.7
**A_{2B} *(h)* (antagonist effect)**					
100043705-1	S-CE-123	2.8E-06 M	-20.5	-3.0	-11.8
**A_3 *(h)* (antagonist effect)**					
100043705-1	S-CE-123	2.8E-06 M	-6.0	2.3	-1.8
**α_{1A} *(h)* (antagonist effect)**					
100043705-1	S-CE-123	2.8E-06 M	4.3	1.9	3.1
**α_{1B} *(h)* (antagonist effect)**					
100043705-1	S-CE-123	2.8E-06 M	-25.8	-4.5	-15.1
**alpha $_{1D}$ *(h)* (antagonist effect)**					
100043705-1	S-CE-123	2.8E-06 M	-2.4	-2.5	-2.4
**α2A *(h)* (antagonist effect)**					
100043705-1	S-CE-123	2.8E-06 M	-7.6	-9.2	-8.4
**α_{2B} *(h)* (antagonist effect)**					
100043705-1	S-CE-123	2.8E-06 M	10.4	-27.0	-8.3
**α_{2C} *(h)* (antagonist effect)**					
100043705-1	S-CE-123	2.8E-06 M	-0.6	1.2	0.3
**β_1 *(h)* (antagonist effect)**					
100043705-1	S-CE-123	2.8E-06 M	28.0	8.9	18.4
**β_2 *(h)* (antagonist effect)**					
100043705-1	S-CE-123	2.8E-06 M	-10.2	7.9	-1.2
**β_3 *(h)* (antagonist effect)**					
100043705-1	S-CE-123	2.8E-06 M	22.9	8.1	15.5
**AT_1 *(h)* (antagonist effect)**					
100043705-1	S-CE-123	2.8E-06 M	6.5	-0.6	3.0
**APJ (apelin) *(h)* (antagonist effect)**					
100043705-1	S-CE-123	2.8E-06 M	-0.9	13.2	6.2
**TGR5 (h) (antagonist effect)**					
100043705-1	S-CE-123	2.8E-06 M	-1.7	1.0	-0.3
**BB_1 *(h)* (antagonist effect)**					
100043705-1	S-CE-123	2.8E-06 M	-14.8	-6.2	-10.5
**BB_2 *(h)* (antagonist effect)**					
100043705-1	S-CE-123	2.8E-06 M	-29.2	-17.3	-23.2
**BB_3 *(h)* (antagonist effect)**					
100043705-1	S-CE-123	2.8E-06 M	3.2	9.2	6.2
**B_1 *(h)* (antagonist effect)**					
100043705-1	S-CE-123	2.8E-06 M	-2.8	-18.1	-10.4
**B_2 *(h)* (antagonist effect)**					
100043705-1	S-CE-123	2.8E-06 M	-23.2	-41.0	-32.1
**CGRP *(h)* (antagonist effect)**					
100043705-1	S-CE-123	2.8E-06 M	-37.7	0.2	-18.8
**CT (Calcitonin) *(h)* (antagonist effect)**					
100043705-1	S-CE-123	2.8E-06 M	-36.5	-6.9	-21.7
**CaS *(h)* (antagonist effect)**					
100043705-1	S-CE-123	2.8E-06 M	-7.9	-4.9	-6.4
**CB_1 *(h)* (antagonist effect)**					
100043705-1	S-CE-123	2.8E-06 M	-0.3	-3.9	-2.1
**CB_2 *(h)* (antagonist effect)**					
100043705-1	S-CE-123	2.8E-06 M	8.6	8.6	8.6
**CCR1 *(h)* (antagonist effect)**					
100043705-1	S-CE-123	2.8E-06 M	-3.7	0.7	-1.5
**CCR2 *(h)* (antagonist effect)**					
100043705-1	S-CE-123	2.8E-06 M	3.1	1.0	2.0

Compound I.D.	Client Compound I.D.	Test Concentration	% Inhibition of Control Agonist Response 1st	2nd	Mean
**CCR3 *(h)* (antagonist effect)**					
100043705-1	S-CE-123	2.8E-06 M	-11.7	-9.6	-10.7
**CCR4 *(h)* (antagonist effect)**					
100043705-1	S-CE-123	2.8E-06 M	-5.5	-6.1	-5.8
**CCR5 (antagonist effect)**					
100043705-1	S-CE-123	2.8E-06 M	18.7	34.7	26.7
**CCR6 *(h)* (antagonist effect)**					
100043705-1	S-CE-123	2.8E-06 M	-33.1	-20.3	-26.7
**CCR7 *(h)* (antagonist effect)**					
100043705-1	S-CE-123	2.8E-06 M	-4.0	9.6	2.8
**CCR8 *(h)* (antagonist effect)**					
100043705-1	S-CE-123	2.8E-06 M	-20.5	-19.2	-19.9
**CCR10 *(h)* (antagonist effect)**					
100043705-1	S-CE-123	2.8E-06 M	-14.8	0.9	-6.9
**CX3CR1 *(h)* (antagonist effect)**					
100043705-1	S-CE-123	2.8E-06 M	-17.2	-14.5	-15.8
**CXCR1 *(h)* (antagonist effect)**					
100043705-1	S-CE-123	2.8E-06 M	-6.6	-19.5	-13.0
**CXCR2 *(h)* (antagonist effect)**					
100043705-1	S-CE-123	2.8E-06 M	-0.2	7.9	3.8
**CXCR3 (h) (antagonist effect)**					
100043705-1	S-CE-123	2.8E-06 M	-9.4	-6.1	-7.8
**CXCR4 *(h)* (antagonist effect)**					
100043705-1	S-CE-123	2.8E-06 M	-62.8	-18.6	-40.7
**CXCR5 *(h)* (antagonist effect)**					
100043705-1	S-CE-123	2.8E-06 M	-0.8	-3.0	-1.9
**CXCR6 *(h)* (antagonist effect)**					
100043705-1	S-CE-123	2.8E-06 M	-10.8	-4.4	-7.6
**CCK_1 (CCK_A) *(h)* (antagonist effect)**					
100043705-1	S-CE-123	2.8E-06 M	28.8	7.7	18.2
**CCK_2 (CCK_B) *(h)* (antagonist effect)**					
100043705-1	S-CE-123	2.8E-06 M	-19.7	5.9	-6.9
**C3aR *(h)* (antagonist effect)**					
100043705-1	S-CE-123	2.8E-06 M	2.3	20.5	11.4
**ChemR23 *(h)* (antagonist effect)**					
100043705-1	S-CE-123	2.8E-06 M	-5.3	2.0	-1.6
**CRF_1 *(h)* (antagonist effect)**					
100043705-1	S-CE-123	2.8E-06 M	-13.8	-19.9	-16.9
**$CRF_{2\alpha}$ *(h)* (antagonist effect)**					
100043705-1	S-CE-123	2.8E-06 M	5.7	36.6	21.1
**XCR1 / GPR5 *(h)* (antagonist effect)**					
100043705-1	S-CE-123	2.8E-06 M	-15.3	-3.1	-9.2
**D_1 *(h)* (antagonist effect)**					
100043705-1	S-CE-123	2.8E-06 M	-15.8	4.6	-5.6
**D_{2S} *(h)* (antagonist effect)**					
100043705-1	S-CE-123	2.8E-06 M	23.8	12.5	18.1
**D_3 *(h)* (antagonist effect)**					
100043705-1	S-CE-123	2.8E-06 M	7.1	-10.9	-1.9
**$D_{4.4}$ *(h)* (antagonist effect)**					
100043705-1	S-CE-123	2.8E-06 M	-9.6	-6.1	-7.9
**D_5 *(h)* (antagonist effect)**					
100043705-1	S-CE-123	2.8E-06 M	11.0	-32.6	-10.8
**ET_A *(h)* (antagonist effect)**					
100043705-1	S-CE-123	2.8E-06 M	-3.3	2.4	-0.5
**ET_B *(h)* (antagonist effect)**					
100043705-1	S-CE-123	2.8E-06 M	-4.6	7.6	1.5

Compound I.D.	Client Compound I.D.	Test Concentration	% Inhibition of Control Agonist Response 1st	2nd	Mean
**FFA1 *(h)* (GPR40) (antagonist effect)**					
100043705-1	S-CE-123	2.8E-06 M	-1.5	-4.1	-2.8
**FFA2 *(h)* (GPR43) (antagonist effect)**					
100043705-1	S-CE-123	2.8E-06 M	1.2	4.6	2.9
**FFA3 *(h)* (GPR41) (antagonist effect)**					
100043705-1	S-CE-123	2.8E-06 M	-0.9	-1.8	-1.4
**FFA4 (GPR120) *(h)* (antagonist effect)**					
100043705-1	S-CE-123	2.8E-06 M	-12.9	-15.9	-14.4
**GnRH *(h)* (antagonist effect)**					
100043705-1	S-CE-123	2.8E-06 M	4.4	3.4	3.9
**GPR39 *(h)* (antagonist effect)**					
100043705-1	S-CE-123	2.8E-06 M	8.6	-5.3	1.6
**OXGR1 GPR99 *(h)* (antagonist effect)**					
100043705-1	S-CE-123	2.8E-06 M	-7.4	-12.9	-10.2
**GPR103/QRFP *(h)* (antagonist effect)**					
100043705-1	S-CE-123	2.8E-06 M	-10.3	-7.4	-8.8
**GPR109A *(h)* (antagonist effect)**					
100043705-1	S-CE-123	2.8E-06 M	-13.5	-9.8	-11.6
**GPR119 (antagonist effect)**					
100043705-1	S-CE-123	2.8E-06 M	-9.8	-1.8	-5.8
**FPR1 *(h)* (antagonist effect)**					
100043705-1	S-CE-123	2.8E-06 M	3.3	-0.1	1.6
**GABAB1b beta *(h)* (antagonist effect)**					
100043705-1	S-CE-123	2.8E-06 M	1.1	-22.3	-10.6
**GAL1 *(h)* (antagonist effect)**					
100043705-1	S-CE-123	2.8E-06 M	0.8	3.9	2.3
**GAL_2 *(h)* (antagonist effect)**					
100043705-1	S-CE-123	2.8E-06 M	-9.0	-0.5	-4.8
**GIP *(h)* (antagonist effect)**					
100043705-1	S-CE-123	2.8E-06 M	-6.2	-7.0	-6.6
**GLP-1 (antagonist effect)**					
100043705-1	S-CE-123	2.8E-06 M	-27.8	-37.0	-32.4
**secretin *(h)* (antagonist effect)**					
100043705-1	S-CE-123	2.8E-06 M	5.7	29.6	17.6
**TSH *(h)* (antagonist effect)**					
100043705-1	S-CE-123	2.8E-06 M	8.9	-21.2	-6.1
**GHRH *(h)* (antagonist effect)**					
100043705-1	S-CE-123	2.8E-06 M	28.8	-5.4	11.7
**Ghrelin / GHSR-1a *(h)* (antagonist effect)**					
100043705-1	S-CE-123	2.8E-06 M	-14.6	-8.7	-11.7
**H_1 *(h)* (antagonist effect)**					
100043705-1	S-CE-123	2.8E-06 M	-1.0	-2.8	-1.9
**H_2 *(h)* (antagonist effect)**					
100043705-1	S-CE-123	2.8E-06 M	-2.7	-2.0	-2.4
**H_3 *(h)* (antagonist effect)**					
100043705-1	S-CE-123	2.8E-06 M	-1.4	0.8	-0.3
**H_4 *(h)* (antagonist effect)**					
100043705-1	S-CE-123	2.8E-06 M	4.4	2.4	3.4
**KiSS1 / GPR54 *(h)* (antagonist effect)**					
100043705-1	S-CE-123	2.8E-06 M	-3.5	-2.0	-2.8
**BLT_1 (LTB_4) *(h)* (antagonist effect)**					
100043705-1	S-CE-123	2.8E-06 M	-4.9	-4.6	-4.7
**$CysLT_1$ (LTD_4) *(h)* (antagonist effect)**					
100043705-1	S-CE-123	2.8E-06 M	-9.2	-16.7	-12.9
**$CysLT_2$ (LTC_4) *(h)* (antagonist effect)**					
100043705-1	S-CE-123	2.8E-06 M	4.2	4.8	4.5

Compound I.D.	Client Compound I.D.	Test Concentration	% Inhibition of Control Agonist Response		
			1st	2nd	Mean
**LPA1(h) (antagonist effect)**					
100043705-1	S-CE-123	2.8E-06 M	-0.4	9.2	4.4
**LPA_2 *(h)* (antagonist effect)**					
100043705-1	S-CE-123	2.8E-06 M	-12.7	-9.1	-10.9
**LPA_3 *(h)* (antagonist effect)**					
100043705-1	S-CE-123	2.8E-06 M	-22.3	-46.4	-34.4
**S_1P_1 *(h)* (antagonist effect)**					
100043705-1	S-CE-123	2.8E-06 M	5.0	6.7	5.9
**S_1P_2 *(h)* (antagonist effect)**					
100043705-1	S-CE-123	2.8E-06 M	-10.4	-0.1	-5.2
**S_1P_3 *(h)* (antagonist effect)**					
100043705-1	S-CE-123	2.8E-06 M	13.9	10.5	12.2
**S1P4 *(h)* (antagonist effect)**					
100043705-1	S-CE-123	2.8E-06 M	-14.2	-14.9	-14.6
**S1P5 *(h)* (antagonist effect)**					
100043705-1	S-CE-123	2.8E-06 M	10.1	-2.8	3.7
**MCH_1 *(h)* (antagonist effect)**					
100043705-1	S-CE-123	2.8E-06 M	-1.7	-0.2	-0.9
**MCH_2 *(h)* (antagonist effect)**					
100043705-1	S-CE-123	2.8E-06 M	1.8	4.4	3.1
**MC_1 (antagonist effect)**					
100043705-1	S-CE-123	2.8E-06 M	-27.7	-24.3	-26.0
**MC_2 *(h)* (antagonist effect)**					
100043705-1	S-CE-123	2.8E-06 M	-0.1	6.2	3.1
**MC_3 *(h)* (antagonist effect)**					
100043705-1	S-CE-123	2.8E-06 M	-25.9	10.3	-7.8
**MC_4 *(h)* (antagonist effect)**					
100043705-1	S-CE-123	2.8E-06 M	-11.0	15.7	2.3
**MC_5 *(h)* (antagonist effect)**					
100043705-1	S-CE-123	2.8E-06 M	5.8	-23.6	-8.9
**MT_1 (ML_{1A}) *(h)* (antagonist effect)**					
100043705-1	S-CE-123	2.8E-06 M	7.6	7.6	7.6
**MT_2 (ML_{1B}) *(h)* (antagonist effect)**					
100043705-1	S-CE-123	2.8E-06 M	2.3	10.2	6.2
**motilin *(h)* (antagonist effect)**					
100043705-1	S-CE-123	2.8E-06 M	-9.5	-0.9	-5.2
**M_1 *(h)* (antagonist effect)**					
100043705-1	S-CE-123	2.8E-06 M	2.0	3.2	2.6
**M_2 *(h)* (antagonist effect)**					
100043705-1	S-CE-123	2.8E-06 M	-3.4	-15.0	-9.2
**M_3 *(h)* (antagonist effect)**					
100043705-1	S-CE-123	2.8E-06 M	4.1	-2.5	0.8
**M_4 *(h)* (antagonist effect)**					
100043705-1	S-CE-123	2.8E-06 M	-8.8	-8.2	-8.5
**M_5 *(h)* (antagonist effect)**					
100043705-1	S-CE-123	2.8E-06 M	-5.2	-6.6	-5.9
**MrgD *(h)* (antagonist effect)**					
100043705-1	S-CE-123	2.8E-06 M	26.9	33.0	30.0
**MRGX1 / MRGPRX1 *(h)* (antagonist effect)**					
100043705-1	S-CE-123	2.8E-06 M	6.4	3.4	4.9
**MRGX2 / MGRPLX2 *(h)* (antagonist effect)**					
100043705-1	S-CE-123	2.8E-06 M	9.6	4.5	7.1
**NPS *(h)* (antagonist effect)**					
100043705-1	S-CE-123	2.8E-06 M	-13.9	-14.7	-14.3
**NPBW1 *(h)* (antagonist effect)**					
100043705-1	S-CE-123	2.8E-06 M	0.1	-4.0	-1.9

Compound I.D.	Client Compound I.D.	Test Concentration	% Inhibition of Control Agonist Response 1st	2nd	Mean
**NK1 *(h)* (antagonist effect)**					
100043705-1	S-CE-123	2.8E-06 M	-11.5	1.0	-5.3
**NK2 *(h)* (antagonist effect)**					
100043705-1	S-CE-123	2.8E-06 M	-1.4	-0.2	-0.8
**NK3 *(h)* (antagonist effect)**					
100043705-1	S-CE-123	2.8E-06 M	-10.2	-0.1	-5.1
**NMU1 *(h)* (antagonist effect)**					
100043705-1	S-CE-123	2.8E-06 M	-25.8	-16.8	-21.3
**NMU2 *(h)* (antagonist effect)**					
100043705-1	S-CE-123	2.8E-06 M	-17.1	-18.4	-17.7
**NTS1 (NT1) *(h)* (antagonist effect)**					
100043705-1	S-CE-123	2.8E-06 M	-19.0	-1.0	-10.0
**NTS2 (NT2) *(h)* (antagonist effect)**					
100043705-1	S-CE-123	2.8E-06 M	26.2	19.6	22.9
**δ (DOP) *(h)* (antagonist effect)**					
100043705-1	S-CE-123	2.8E-06 M	-2.2	-3.3	-2.8
**κ (KOP) (antagonist effect)**					
100043705-1	S-CE-123	2.8E-06 M	12.8	2.8	7.8
**μ (MOP) *(h)* (antagonist effect)**					
100043705-1	S-CE-123	2.8E-06 M	-2.2	12.2	5.0
**NOP *(h)* (ORL1) (antagonist effect)**					
100043705-1	S-CE-123	2.8E-06 M	19.3	17.6	18.4
**OX1 *(h)* (antagonist effect)**					
100043705-1	S-CE-123	2.8E-06 M	-16.5	-12.5	-14.5
**OX2 *(h)* (antagonist effect)**					
100043705-1	S-CE-123	2.8E-06 M	3.7	4.7	4.2
**PAF *(h)* (antagonist effect)**					
100043705-1	S-CE-123	2.8E-06 M	-38.5	-39.8	-39.1
**PTH1 *(h)* (antagonist effect)**					
100043705-1	S-CE-123	2.8E-06 M	-11.0	-10.2	-10.6
**PK1 *(h)* (antagonist effect)**					
100043705-1	S-CE-123	2.8E-06 M	12.9	-7.9	2.5
**PK2 *(h)* (antagonist effect)**					
100043705-1	S-CE-123	2.8E-06 M	3.5	11.1	7.3
**DP1 *(h)* (antagonist effect)**					
100043705-1	S-CE-123	2.8E-06 M	-9.7	4.7	-2.5
**EP1 *(h)* (antagonist effect)**					
100043705-1	S-CE-123	2.8E-06 M	-13.9	-9.8	-11.9
**EP2 *(h)* (antagonist effect)**					
100043705-1	S-CE-123	2.8E-06 M	-11.0	12.3	0.7
**EP3 *(h)* (antagonist effect)**					
100043705-1	S-CE-123	2.8E-06 M	-33.0	-28.7	-30.8
**EP4 *(h)* (antagonist effect)**					
100043705-1	S-CE-123	2.8E-06 M	-2.8	-0.3	-1.5
**FP *(h)* (antagonist effect)**					
100043705-1	S-CE-123	2.8E-06 M	7.8	5.6	6.7
**IP (PGI2) *(h)* (antagonist effect)**					
100043705-1	S-CE-123	2.8E-06 M	23.9	8.7	16.3
**TP (TXA2/PGH2) *(h)* (antagonist effect)**					
100043705-1	S-CE-123	2.8E-06 M	0.7	4.3	2.5
**PAR1 *(h)* (antagonist effect)**					
100043705-1	S-CE-123	2.8E-06 M	1.3	3.0	2.1
**PAR2 *(h)* (antagonist effect)**					
100043705-1	S-CE-123	2.8E-06 M	-17.9	-20.9	-19.4
**P2Y1 *(h)* (antagonist effect)**					
100043705-1	S-CE-123	2.8E-06 M	-6.6	-6.8	-6.7

Compound I.D.	Client Compound I.D.	Test Concentration	% Inhibition of Control Agonist Response 1st	2nd	Mean
**$P2Y_2$ *(h)* (antagonist effect)**					
100043705-1	S-CE-123	2.8E-06 M	-29.4	-12.2	-20.8
**$P2Y_4$ *(h)* (antagonist effect)**					
100043705-1	S-CE-123	2.8E-06 M	-3.2	-3.8	-3.5
**$P2Y_6$ *(h)* (antagonist effect)**					
100043705-1	S-CE-123	2.8E-06 M	5.6	1.8	3.7
**P2Y11 *(h)* (antagonist effect)**					
100043705-1	S-CE-123	2.8E-06 M	2.9	-4.1	-0.6
**PTH2 *(h)* (antagonist effect)**					
100043705-1	S-CE-123	2.8E-06 M	-2.4	-1.3	-1.9
**RXFP1 *(h)* (antagonist effect)**					
100043705-1	S-CE-123	2.8E-06 M	21.4	-19.4	1.0
**5-HT1A *(h)* (antagonist effect)**					
100043705-1	S-CE-123	2.8E-06 M	-9.6	-6.7	-8.2
**5HT1B (h) (antagonist effect)**					
100043705-1	S-CE-123	2.8E-06 M	-12.7	-9.8	-11.3
**$5\text{-}HT_{1D}$ (antagonist effect)**					
100043705-1	S-CE-123	2.8E-06 M	-3.8	1.9	-0.9
**$5\text{-}HT_{2A}$ *(h)* (antagonist effect)**					
100043705-1	S-CE-123	2.8E-06 M	-9.3	-1.2	-5.2
**$5\text{-}HT_{2B}$ *(h)* (antagonist effect)**					
100043705-1	S-CE-123	2.8E-06 M	8.7	-0.6	4.1
**$5\text{-}HT_{2C}$ *(h)* (antagonist effect)**					
100043705-1	S-CE-123	2.8E-06 M	6.4	-5.5	0.4
**$5\text{-}HT_{4e}$ *(h)* (antagonist effect)**					
100043705-1	S-CE-123	2.8E-06 M	34.4	18.8	26.6
**$5\text{-}HT_6$ *(h)* (antagonist effect)**					
100043705-1	S-CE-123	2.8E-06 M	-12.0	14.9	1.4
**$5\text{-}HT_7$ *(h)* (antagonist effect)**					
100043705-1	S-CE-123	2.8E-06 M	2.0	0.5	1.3
**sst_1 *(h)* (antagonist effect)**					
100043705-1	S-CE-123	2.8E-06 M	-9.9	-3.3	-6.6
**sst2 *(h)* (antagonist effect)**					
100043705-1	S-CE-123	2.8E-06 M	1.5	-13.1	-5.8
**sst3 *(h)* (antagonist effect)**					
100043705-1	S-CE-123	2.8E-06 M	-4.5	-6.3	-5.4
**sst_4 *(h)* (antagonist effect)**					
100043705-1	S-CE-123	2.8E-06 M	16.0	2.4	9.2
**sst_5 *(h)* (antagonist effect)**					
100043705-1	S-CE-123	2.8E-06 M	-5.6	2.7	-1.4
**SUCNR1/GPR91 *(h)* (antagonist effect)**					
100043705-1	S-CE-123	2.8E-06 M	-9.9	-6.0	-7.9
**TRH_1 *(h)* (antagonist effect)**					
100043705-1	S-CE-123	2.8E-06 M	3.0	7.2	5.1
**UT *(h)* (antagonist effect)**					
100043705-1	S-CE-123	2.8E-06 M	14.5	10.7	12.6
**PAC_1 (PACAP) *(h)* (antagonist effect)**					
100043705-1	S-CE-123	2.8E-06 M	20.9	38.9	29.9
**$VPAC_1$ (VIP_1) *(h)* (antagonist effect)**					
100043705-1	S-CE-123	2.8E-06 M	19.4	8.5	13.9
**Y4 *(h)* (antagonist effect)**					
100043705-1	S-CE-123	2.8E-06 M	-10.2	-4.2	-7.2
**V_{1a} *(h)* (antagonist effect)**					
100043705-1	S-CE-123	2.8E-06 M	-19.2	-16.1	-17.6
**V1B *(h)* (antagonist effect)**					
100043705-1	S-CE-123	2.8E-06 M	-4.6	-7.1	-5.9
**V_2 *(h)* (antagonist effect)**					
100043705-1	S-CE-123	2.8E-06 M	25.0	15.1	20.1

Table S4 Reference compounds for antagonist effect.

Compound I.D.	IC_{50} (M)	K_B (M)	nH
**A1 *(h)* (antagonist effect)**			
DPCPX	5.1E-09 M	1.2E-09 M	n/a
**A_{2A} (antagonist effect)**			
ZM 241385	6.9E-10 M	7.4E-11 M	n/a
**A_{2B} *(h)* (antagonist effect)**			
XAC	2.2E-07 M	2.7E-08 M	n/a
**A_3 *(h)* (antagonist effect)**			
MRS 1220	6.4E-09 M	3.0E-10 M	n/a
**α_{1A} *(h)* (antagonist effect)**			
WB 4101	1.7E-09 M	2.3E-10 M	n/a
**α_{1B} *(h)* (antagonist effect)**			
L765314	8.5E-09 M	9.1E-10 M	n/a
**alpha $_{1D}$ *(h)* (antagonist effect)**			
WB4101	2.9E-09 M	8.3E-10 M	n/a
**α2A *(h)* (antagonist effect)**			
RX 821002	1.1E-08 M	3.6E-09 M	n/a
**α_{2B} *(h)* (antagonist effect)**			
yohimbine	4.0E-07 M	5.2E-08 M	n/a
**α_{2C} *(h)* (antagonist effect)**			
rauwolscine	3.6E-08 M	1.7E-09 M	n/a
**β_1 *(h)* (antagonist effect)**			
atenolol	2.3E-07 M	2.8E-08 M	n/a
**β_2 *(h)* (antagonist effect)**			
ICI 118551	3.0E-09 M	2.8E-10 M	n/a
**β_3 *(h)* (antagonist effect)**			
cyanopindolol	4.0E-07 M	1.4E-07 M	n/a
**AT_1 *(h)* (antagonist effect)**			
saralasin	1.0E-09 M	8.5E-11 M	n/a
**BB_1 *(h)* (antagonist effect)**			
PD 168368	2.2E-07 M	5.3E-09 M	n/a
**BB_2 *(h)* (antagonist effect)**			
ICI 216,140	5.3E-07 M	6.9E-08 M	n/a
**B_1 *(h)* (antagonist effect)**			
LysdesArg^{9}[Leu^{8}]-BK	1.9E-10 M	4.0E-11 M	n/a
**B_2 *(h)* (antagonist effect)**			
HOE 140	3.1E-08 M	3.6E-09 M	n/a
**CGRP *(h)* (antagonist effect)**			
hCGRPα (8-37)	4.5E-08 M	7.7E-09 M	n/a
**CT (Calcitonin) *(h)* (antagonist effect)**			
salmon calcitonin 8-32	6.9E-08 M	4.1E-09 M	n/a
**CB_1 *(h)* (antagonist effect)**			
AM 281	1.0E-08 M	1.1E-09 M	n/a
**CB_2 *(h)* (antagonist effect)**			
AM 630	7.4E-07 M	4.9E-08 M	n/a
**CCR1 *(h)* (antagonist effect)**			
J113863	6.2E-09 M	1.0E-09 M	n/a
**CCR2 *(h)* (antagonist effect)**			
MIP-II	2.6E-07 M	2.1E-08 M	n/a
**CCR3 *(h)* (antagonist effect)**			
J113863	3.4E-09 M	7.6E-10 M	n/a

Compound I.D.	IC_{50} (M)	K_B (M)	nH
**CXCR2 *(h)* (antagonist effect)**			
SB225002	1.7E-06 M	1.6E-07 M	n/a
**CXCR4 *(h)* (antagonist effect)**			
MIP II	6.5E-08 M	1.6E-09 M	n/a
**CCK_1 (CCK_A) *(h)* (antagonist effect)**			
devazepide	1.1E-10 M	1.2E-11 M	n/a
**CCK_2 (CCK_B) *(h)* (antagonist effect)**			
YM022	6.0E-10 M	7.3E-11 M	n/a
**C3aR *(h)* (antagonist effect)**			
SB290157	2.4E-06 M	8.1E-08 M	n/a
**CRF_1 *(h)* (antagonist effect)**			
antalarmine	9.6E-08 M	3.4E-08 M	n/a
**$CRF_{2\alpha}$ *(h)* (antagonist effect)**			
astressin	4.9E-08 M	4.8E-09 M	n/a
**D_1 *(h)* (antagonist effect)**			
SCH 23390	2.5E-09 M	4.1E-10 M	n/a
**D_{2S} *(h)* (antagonist effect)**			
butaclamol	8.3E-09 M	5.4E-10 M	n/a
**D_3 *(h)* (antagonist effect)**			
(+)butaclamol	1.2E-08 M	1.5E-09 M	n/a
**$D_{4.4}$ *(h)* (antagonist effect)**			
clozapine	1.2E-07 M	1.5E-08 M	n/a
**D_5 *(h)* (antagonist effect)**			
SCH 23390	1.3E-09 M	3.9E-10 M	n/a
**ET_A *(h)* (antagonist effect)**			
BQ-123	3.3E-10 M	3.0E-11 M	n/a
**ET_B *(h)* (antagonist effect)**			
BQ-788	3.6E-08 M	9.6E-09 M	n/a
**GnRH *(h)* (antagonist effect)**			
Cetrorelix	3.0E-09 M	4.8E-10 M	n/a
**GABAB1b beta *(h)* (antagonist effect)**			
CGP55845A	2.5E-09 M	2.6E-10 M	n/a
**GLP-1 (antagonist effect)**			
exendin-3(9-39)	3.1E-09 M	5.9E-10 M	n/a
**GHRH *(h)* (antagonist effect)**			
[N-Acetyl-Tyr^1,D-Arg^2]-GHRF	2.1E-08 M	4.6E-09 M	n/a
**Ghrelin / GHSR-1a *(h)* (antagonist effect)**			
Substance P	3.8E-08 M	7.1E-09 M	n/a
**H_1 *(h)* (antagonist effect)**			
pyrilamine	6.7E-09 M	1.6E-09 M	n/a
**H_2 *(h)* (antagonist effect)**			
cimetidine	5.9E-06 M	9.1E-07 M	n/a
**H_3 *(h)* (antagonist effect)**			
thioperamide	2.1E-06 M	6.1E-08 M	n/a
**H_4 *(h)* (antagonist effect)**			
JNJ 7777120	3.9E-08 M	2.3E-09 M	n/a
**$CysLT_1$ (LTD_4) *(h)* (antagonist effect)**			
MK 571	4.2E-10 M	9.7E-11 M	n/a
**LPA1(h) (antagonist effect)**			
KI16425	7.6E-08 M	6.9E-09 M	n/a
**S_1P_1 *(h)* (antagonist effect)**			
VPC23019	5.1E-08 M	1.1E-08 M	n/a
**S_1P_3 *(h)* (antagonist effect)**			
JTE 013	4.8E-09 M	1.0E-09 M	n/a
**MC_1 (antagonist effect)**			
AGRP (83-132)	2.5E-07 M	9.3E-09 M	n/a
**MC_3 *(h)* (antagonist effect)**			
SHU 9119	3.4E-08 M	6.4E-10 M	n/a

Compound I.D.	IC_{50} (M)	K_B (M)	nH
**MC_4 *(h)* (antagonist effect)**			
SHU 9119	7.3E-10 M	7.5E-11 M	n/a
**MC_5 *(h)* (antagonist effect)**			
AGRP (83-132)	1.4E-07 M	3.9E-08 M	n/a
**MT_1 (ML_{1A}) *(h)* (antagonist effect)**			
luzindole	1.8E-06 M	1.7E-07 M	n/a
**M_1 *(h)* (antagonist effect)**			
pirenzepine	2.9E-08 M	3.4E-09 M	n/a
**M_2 *(h)* (antagonist effect)**			
methoctramine	1.4E-07 M	1.6E-08 M	n/a
**M_3 *(h)* (antagonist effect)**			
4-DAMP	5.2E-09 M	4.7E-10 M	n/a
**M_4 *(h)* (antagonist effect)**			
PD 102807	9.3E-08 M	8.4E-09 M	n/a
**M_5 *(h)* (antagonist effect)**			
Atropine	2.4E-09 M	2.2E-10 M	n/a
**NPS *(h)* (antagonist effect)**			
SHA68	4.7E-08 M	1.3E-09 M	n/a
**NK_1 *(h)* (antagonist effect)**			
L 733,060	1.2E-09 M	1.8E-10 M	n/a
**NK_2 *(h)* (antagonist effect)**			
GR 159897	8.4E-10 M	1.1E-10 M	n/a
**NK_3 *(h)* (antagonist effect)**			
SB 222200	4.2E-08 M	3.8E-09 M	n/a
**NTS_1 (NT_1) *(h)* (antagonist effect)**			
SR142948	2.9E-08 M	2.6E-09 M	n/a
**NTS_2 (NT2) *(h)* (antagonist effect)**			
neurotensin	2.3E-07 M	4.6E-08 M	n/a
**δ (DOP) *(h)* (antagonist effect)**			
Naltriben	7.7E-09 M	1.1E-09 M	n/a
**κ (KOP) (antagonist effect)**			
nor-BNI	7.8E-10 M	1.3E-10 M	n/a
**μ (MOP) *(h)* (antagonist effect)**			
CTOP	7.9E-08 M	7.2E-09 M	n/a
**NOP *(h)* (ORL1) (antagonist effect)**			
UFP-101	1.5E-07 M	4.0E-08 M	n/a
**OX_1 *(h)* (antagonist effect)**			
SB 334867	5.8E-08 M	1.2E-08 M	n/a
**OX_2 *(h)* (antagonist effect)**			
JNJ 10397049	9.5E-08 M	1.3E-08 M	n/a
**PTH1 *(h)* (antagonist effect)**			
PTHrP (7-34)	2.2E-07 M	2.8E-08 M	n/a
**DP_1 *(h)* (antagonist effect)**			
MK0524	1.8E-09 M	3.5E-10 M	n/a
**EP_1 *(h)* (antagonist effect)**			
SC 51322	2.6E-08 M	4.1E-09 M	n/a
**EP_2 *(h)* (antagonist effect)**			
AH 6809	2.7E-06 M	8.6E-07 M	n/a
**EP3 *(h)* (antagonist effect)**			
L798,106	4.4E-10 M	7.3E-11 M	n/a
**EP_4 *(h)* (antagonist effect)**			
GW627368X	7.7E-08 M	7.3E-09 M	n/a
**IP (PGI_2) *(h)* (antagonist effect)**			
CAY10441	3.3E-08 M	4.6E-09 M	n/a
**TP (TXA_2/PGH_2) *(h)* (antagonist effect)**			
L 670596	1.4E-09 M	2.0E-10 M	n/a
**PAR1 *(h)* (antagonist effect)**			
SCH 79797	1.8E-06 M	2.5E-07 M	n/a

Compound I.D.	IC_{50} (M)	K_B (M)	nH
**P2Y1 *(h)* (antagonist effect)**			
MRS 2500	1.9E-09 M	4.3E-10 M	n/a
**$P2Y_2$ *(h)* (antagonist effect)**			
AR-C 118925XX	3.9E-07 M	6.6E-08 M	n/a
**5-HT1A *(h)* (antagonist effect)**			
(S)-WAY-100635	5.2E-09 M	1.1E-09 M	n/a
**5HT1B (h) (antagonist effect)**			
GR55562	6.9E-08 M	1.1E-08 M	n/a
**$5\text{-}HT_{1D}$ (antagonist effect)**			
methiothepin	1.7E-07 M	1.1E-08 M	n/a
**$5\text{-}HT_{2A}$ *(h)* (antagonist effect)**			
ketanserin	6.5E-09 M	1.3E-09 M	n/a
**$5\text{-}HT_{2B}$ *(h)* (antagonist effect)**			
SB 206553	1.9E-08 M	2.9E-09 M	n/a
**$5\text{-}HT_{2C}$ *(h)* (antagonist effect)**			
SB 206553	1.3E-08 M	2.9E-09 M	n/a
**$5\text{-}HT_{4e}$ *(h)* (antagonist effect)**			
GR 113808	3.8E-10 M	5.6E-11 M	n/a
**$5\text{-}HT_6$ *(h)* (antagonist effect)**			
methiothepin	6.3E-09 M	9.8E-10 M	n/a
**$5\text{-}HT_7$ *(h)* (antagonist effect)**			
methiothepin	1.1E-09 M	1.3E-10 M	n/a
**PAC_1 (PACAP) *(h)* (antagonist effect)**			
$PACAP_{6\text{-}38}$	2.1E-08 M	7.8E-09 M	n/a
**$VPAC_1$ (VIP_1) *(h)* (antagonist effect)**			
VIP $GRF_{8\text{-}27}$	9.0E-08 M	1.8E-08 M	n/a
**V_{1a} *(h)* (antagonist effect)**			
$[d(CH_2)_5^{\ 1},Tyr(Me)_2]$-AVP	5.3E-09 M	1.8E-10 M	n/a
**V_2 *(h)* (antagonist effect)**			
[adamantaneacetyl^1,O-Et-D-Tyr^2,Val^4,aminobutyryl^6]-AVP	3.0E-08 M	9.1E-09 M	n/a

Kinase binding assays

This study was outsourced to DiscoverX (KINOMEscan®, Freemont, CA, USA) and is compliant with the corresponding project BUY004-01-p-00001.

Table S5 A list of the 450 protein and lipid kinases representative of all major human kinase families tested as potential targets of S-CE-123 at 2.8E-06 M (IC_{50} for DAT re-uptake inhibition) *in vitro* binding assay. Results are reported as percent control (% Ctrl), where lower numbers indicate stronger hits in the matrix on the following page(s). %Ctrl calculation: ((test compound signal-positive control signal) / (negative control signal – positive control signal)) x 100.

Target	S-CE-123
Gene Symbol	%Ctrl @ 2800nM
AAK1	88
ABL1(E255K)-phosphorylated	73
ABL1(F317I)-nonphosphorylated	100
ABL1(F317I)-phosphorylated	92
ABL1(F317L)-nonphosphorylated	100
ABL1(F317L)-phosphorylated	100
ABL1(H396P)-nonphosphorylated	86
ABL1(H396P)-phosphorylated	90
ABL1(M351T)-phosphorylated	100
ABL1(Q252H)-nonphosphorylated	66
ABL1(Q252H)-phosphorylated	84
ABL1(T315I)-nonphosphorylated	100
ABL1(T315I)-phosphorylated	98
ABL1(Y253F)-phosphorylated	93
ABL1-nonphosphorylated	93
ABL1-phosphorylated	100
ABL2	99
ACVR1	99
ACVR1B	100
ACVR2A	100
ACVR2B	100
ACVRL1	84
ADCK3	87
ADCK4	96
AKT1	99
AKT2	87
AKT3	99
ALK	100
ALK(C1156Y)	100
ALK(L1196M)	100
AMPK-alpha1	92
AMPK-alpha2	97
ANKK1	100
ARK5	100
ASK1	84
ASK2	100
AURKA	91
AURKB	78
AURKC	100
AXL	96
BIKE	97
BLK	90
BMPR1A	100
BMPR1B	100
BMPR2	100
BMX	57
BRAF	100
BRAF(V600E)	100

Target	S-CE-123
Gene Symbol	%Ctrl @ 2800nM
BRK	96
BRSK1	100
BRSK2	100
BTK	100
BUB1	100
CAMK1	97
CAMK1B	100
CAMK1D	96
CAMK1G	82
CAMK2A	93
CAMK2B	87
CAMK2D	88
CAMK2G	95
CAMK4	91
CAMKK1	95
CAMKK2	94
CASK	82
CDC2L1	95
CDC2L2	98
CDC2L5	98
CDK11	71
CDK2	94
CDK3	100
CDK4	100
CDK4-cyclinD1	100
CDK4-cyclinD3	85
CDK5	97
CDK7	70
CDK8	96
CDK9	92
CDKL1	100
CDKL2	89
CDKL3	81
CDKL5	97
CHEK1	89
CHEK2	78
CIT	74
CLK1	96
CLK2	93
CLK3	95
CLK4	100
CSF1R	76
CSF1R-autoinhibited	100
CSK	98
CSNK1A1	74
CSNK1A1L	100
CSNK1D	100
CSNK1E	96
CSNK1G1	96

Target	S-CE-123
Gene Symbol	%Ctrl @ 2800nM
CSNK1G2	94
CSNK1G3	98
CSNK2A1	88
CSNK2A2	100
CTK	51
DAPK1	91
DAPK2	96
DAPK3	96
DCAMKL1	100
DCAMKL2	97
DCAMKL3	96
DDR1	100
DDR2	100
DLK	100
DMPK	96
DMPK2	88
DRAK1	95
DRAK2	95
DYRK1A	96
DYRK1B	40
DYRK2	86
EGFR	92
EGFR(E746-A750del)	93
EGFR(G719C)	74
EGFR(G719S)	77
EGFR(L747-E749del, A750P)	93
EGFR(L747-S752del, P753S)	96
EGFR(L747-T751del,Sins)	57
EGFR(L858R)	92
EGFR(L858R,T790M)	99
EGFR(L861Q)	98
EGFR(S752-I759del)	66
EGFR(T790M)	98
EIF2AK1	100
EPHA1	96
EPHA2	73
EPHA3	97
EPHA4	97
EPHA5	86
EPHA6	100
EPHA7	96
EPHA8	100
EPHB1	100
EPHB2	89
EPHB3	100
EPHB4	95
EPHB6	89
ERBB2	100
ERBB3	89

%Ctrl Legend

0≤x<.1	.1≤x<1	1≤x<10	10≤x<35	x≥35

Target	S-CE-123
Gene Symbol	%Ctrl @ 2800nM
ERBB4	96
ERK1	91
ERK2	95
ERK3	93
ERK4	100
ERK5	91
ERK8	100
ERN1	86
FAK	100
FER	100
FES	95
FGFR1	100
FGFR2	87
FGFR3	91
FGFR3(G697C)	96
FGFR4	100
FGR	100
FLT1	100
FLT3	96
FLT3(D835H)	58
FLT3(D835V)	100
FLT3(D835Y)	99
FLT3(ITD)	100
FLT3(ITD,D835V)	100
FLT3(ITD,F691L)	50
FLT3(K663Q)	96
FLT3(N841I)	91
FLT3(R834Q)	90
FLT3-autoinhibited	99
FLT4	100
FRK	96
FYN	93
GAK	100
GCN2(Kin.Dom.2,S808G)	100
GRK1	100
GRK2	100
GRK3	100
GRK4	73
GRK7	90
GSK3A	99
GSK3B	92
HASPIN	95
HCK	99
HIPK1	90
HIPK2	100
HIPK3	91
HIPK4	100
HPK1	89
HUNK	100
ICK	91
IGF1R	94
IKK-alpha	100
IKK-beta	96
IKK-epsilon	98
INSR	100
INSRR	94
IRAK1	91
IRAK3	96
IRAK4	94
ITK	100
JAK1(JH1domain-catalytic)	93
JAK1(JH2domain-pseudokinase)	92
JAK2(JH1domain-catalytic)	100
JAK3(JH1domain-catalytic)	100
JNK1	67
JNK2	90
JNK3	74
KIT	97
KIT(A829P)	98
KIT(D816H)	100
KIT(D816V)	97
KIT(L576P)	99
KIT(V559D)	97
KIT(V559D,T670I)	83
KIT(V559D,V654A)	100
KIT-autoinhibited	86
LATS1	93
LATS2	100
LCK	100
LIMK1	98
LIMK2	88
LKB1	100
LOK	95
LRRK2	100
LRRK2(G2019S)	96
LTK	93
LYN	83
LZK	94
MAK	94
MAP3K1	85
MAP3K15	84
MAP3K2	100
MAP3K3	86
MAP3K4	84
MAP4K2	100
MAP4K3	100
MAP4K4	100
MAP4K5	95
MAPKAPK2	99
MAPKAPK5	100
MARK1	93
MARK2	100
MARK3	70
MARK4	98
MAST1	94
MEK1	94
MEK2	93
MEK3	86
MEK4	100
MEK5	98
MEK6	89
MELK	67
MERTK	63
MET	99
MET(M1250T)	99
MET(Y1235D)	99
MINK	87
MKK7	100
MKNK1	96
MKNK2	69
MLCK	96
MLK1	100
MLK2	96
MLK3	100
MRCKA	95
MRCKB	98
MST1	97
MST1R	93
MST2	80
MST3	87
MST4	100
MTOR	100
MUSK	100
MYLK	96
MYLK2	99
MYLK4	89
MYO3A	96
MYO3B	52
NDR1	83
NDR2	82
NEK1	90
NEK10	100
NEK11	100
NEK2	96
NEK3	100
NEK4	83
NEK5	90

%Ctrl Legend

0≤x<.1	.1≤x<1	1≤x<10	10≤x<35	x≥35

Target	S-CE-123
Gene Symbol	%Ctrl @ 2800nM
NEK6	100
NEK7	100
NEK9	100
NIK	100
NIM1	97
NLK	97
OSR1	90
p38-alpha	100
p38-beta	94
p38-delta	95
p38-gamma	100
PAK1	92
PAK2	77
PAK3	70
PAK4	98
PAK6	100
PAK7	98
PCTK1	97
PCTK2	93
PCTK3	99
PDGFRA	98
PDGFRB	99
PDPK1	95
PFCDPK1(P.falciparum)	70
PFPK5(P.falciparum)	84
PFTAIRE2	100
PFTK1	92
PHKG1	100
PHKG2	96
PIK3C2B	98
PIK3C2G	66
PIK3CA	80
PIK3CA(C420R)	92
PIK3CA(E542K)	88
PIK3CA(E545A)	93
PIK3CA(E545K)	82
PIK3CA(H1047L)	100
PIK3CA(H1047Y)	100
PIK3CA(I800L)	84
PIK3CA(M1043I)	63
PIK3CA(Q546K)	97
PIK3CB	93
PIK3CD	86
PIK3CG	70
PIK4CB	94
PIKFYVE	100
PIM1	100
PIM2	100
PIM3	100

Target	S-CE-123
Gene Symbol	%Ctrl @ 2800nM
PIP5K1A	95
PIP5K1C	100
PIP5K2B	100
PIP5K2C	100
PKAC-alpha	95
PKAC-beta	100
PKMYT1	88
PKN1	100
PKN2	86
PKNB(M.tuberculosis)	96
PLK1	79
PLK2	89
PLK3	95
PLK4	89
PRKCD	89
PRKCE	100
PRKCH	100
PRKCI	100
PRKCQ	100
PRKD1	92
PRKD2	98
PRKD3	96
PRKG1	100
PRKG2	99
PRKR	93
PRKX	100
PRP4	92
PYK2	100
QSK	93
RAF1	76
RET	96
RET(M918T)	89
RET(V804L)	91
RET(V804M)	95
RIOK1	77
RIOK2	99
RIOK3	97
RIPK1	80
RIPK2	100
RIPK4	97
RIPK5	95
ROCK1	100
ROCK2	100
ROS1	91
RPS6KA4(Kin.Dom.1-N-terminal)	100
RPS6KA4(Kin.Dom.2-C-terminal)	99
RPS6KA5(Kin.Dom.1-N-terminal)	100
RPS6KA5(Kin.Dom.2-C-terminal)	85
RSK1(Kin.Dom.1-N-terminal)	80

Target	S-CE-123
Gene Symbol	%Ctrl @ 2800nM
RSK1(Kin.Dom.2-C-terminal)	92
RSK2(Kin.Dom.1-N-terminal)	100
RSK2(Kin.Dom.2-C-terminal)	96
RSK3(Kin.Dom.1-N-terminal)	100
RSK3(Kin.Dom.2-C-terminal)	87
RSK4(Kin.Dom.1-N-terminal)	100
RSK4(Kin.Dom.2-C-terminal)	94
S6K1	90
SBK1	100
SGK	94
SgK110	72
SGK2	100
SGK3	100
SIK	100
SIK2	100
SLK	100
SNARK	100
SNRK	98
SRC	100
SRMS	99
SRPK1	92
SRPK2	76
SRPK3	76
STK16	97
STK33	80
STK35	98
STK36	88
STK39	100
SYK	100
TAK1	71
TAOK1	100
TAOK2	100
TAOK3	91
TBK1	100
TEC	100
TESK1	96
TGFBR1	86
TGFBR2	88
TIE1	100
TIE2	100
TLK1	91
TLK2	95
TNIK	89
TNK1	74
TNK2	100
TNNI3K	100
TRKA	100
TRKB	100
TRKC	100

%Ctrl Legend

0≤x<.1	.1≤x<1	1≤x<10	10≤x<35	x≥35

Target	S-CE-123
Gene Symbol	%Ctrl @ 2800nM
TRPM6	100
TSSK1B	73
TSSK3	100
TTK	86
TXK	89
TYK2(JH1domain-catalytic)	98
TYK2(JH2domain-pseudokinase)	100
TYRO3	100
ULK1	100
ULK2	100
ULK3	100
VEGFR2	97
VPS34	100
VRK2	98
WEE1	100
WEE2	100
WNK1	68
WNK2	100
WNK3	100
WNK4	100
YANK1	88
YANK2	98
YANK3	100
YES	100
YSK1	88
YSK4	96
ZAK	88
ZAP70	96

%Ctrl Legend

$0 \le x < .1$	$.1 \le x < 1$	$1 \le x < 10$	$10 \le x < 35$	$x \ge 35$

Pharmacokinetics study in rats after single intra-peritoneal administration of *S*-CE-123 at 10 mg/kg/body weight

Animals

Eighteen male Sprague Dawley (Rj Han:SD) rats

Age/weight: 200-225 grams at the day of the delivery day

Breeder: Janvier Labs , Le Genest-Saint-Isle, 53940 Saint Berthevin Cedex, France.

Pre-treatment period: after their arrival, the animals were acclimated for a period of at least 5 days before the beginning of the surgery or the dosing period.

From animal arrival at Evotec France, the animals were group-housed by 2 corresponding to the same compound treatment in dedicated home cages (1291H; size 820 cm2 , Tecniplast);

The animal room conditions were set as follow: temperature: 22 ± 2°C, relative humidity : 50 ± 10%, light/dark cycle : 12 h/12 h, air change rate : 12 to 15 cycles/hour of filtered, 100% fresh air. All animals

had free access to filtered tap water. All rats are fed with a maintenance chow (AO4C, Safe, 89290 Augy) during the acclimation and the study.

Jugular vein cannula implantation

Materials:

• catheter C30PU-RJV1303 – polyurethane ; size : 3Fr (Ø 0.6X1.0 mm) L : 30cm ;

• pin-port and pin-port injector;

• heparinized saline solution: 5 mL d'Héparine ChoayND (25 000UI corresponding to 5000 UI/mL) in 45 mL of saline solution NaCl 0.9% corresponding to a final concentration of 500 UI/mL

• Isoflurane anesthesia (Isoflo 100%)

• Buprenorphine (Buprecare)

• Disinfectant soap (Polyvidone iode VetedineScrub) and disfectant (10% Vetedine solution)

• Alcohol 70°

• 5-0 non absorbable suture with curved needle

• Sterile gauze pads

• Heating pads

• Sterile saline

Methods:

The main step for the implantation of cannula in jugular vein are described as follows

• Remove the hair and disinfect the incision sites (dorsal and ventral) with appropriate disinfection protocol with Vetedine ScrubND, Alcohol 70° and Vetedine ND (repeated 3 times); apply lubricant ointment on the eye.

• Administer buprenorphine (BuprecareND, multidose 0.3 mg/ml, Axience) 0.05 mg/kg by SC route just before surgery and 8 H post-surgery

• Prepare the catheter and fill it in with heparinized saline solution

• Place the rat in dorsal recumbency on the heating pad

• Make a 1 cm ventral cervical skin incision right of the midline of the neck at the level of the clavicle

• Using a hemostat, bluntly dissect the right jugular vein, separate out the salivary and lymphatic tissues to visualize and isolate a 5 mm section of the vessel. Using 5-0 non absorbable suture, place a loose tie on both cranial and caudal ends of the vessel to maximize the exposure of the vessel. Using a micro surgical scissor make an incision large enough to pass the catheter, in line with the vessel between the two ligatures and tie the cranial ligature around the vessel.

• Insert the venous catheter into the vessel towards the heart with the assistance of the micro dissecting hook and forceps and advance the catheter until all of the PU 3F segment is in the vessel. Use the ligatures at the cranial and caudal ends to secure the catheter to the vessel.

• Place the rat in sternal recumbency; make a 1 cm dorsal skin incision at the center of the nape of the neck (between the scapulae).

• Through the ventral incision, a probe with the catheter is guided under the skin to the dorsal incision; the top of the catheter is now connecter to pin-port system.

• After blood flow observed in the PE, close the skin incision with 5-0 non absorbable suture and disinfect with vetedine ND.

Rats are recovered during two days before starting the study.

Table S6 Study design

Group	Number of animals/ Group	Test Article	Dose Level (mg/kg)	Route	Dose Volume (mL/kg)	Study design	Sampling time number	Blood sampling	Brain collection
IP group (serial design) – 10 mg/Kg	3	(S)-CE-123	10	IP	5	Serial	6	Cannula	Yes @ 24 hrs as terminal sampling
IP group (destructive design) – 10 mg/Kg	15	(S)-CE-123	10	IP	5	Destructive	5	Cava vena	Yes

Randomization

Before surgery, the rats have been randomized depending on their weights to obtain groups

of rats with homogeneous weights for surgery.

On the day of dosing, the catheterized rats have also been randomized as well as the rats for

destructive design to obtain groups of three rats with homogeneous weights for the study.

Dose Administration

For intraperitoneal route, the dose formulation were administered by intraperitoneal injection in the rat's lower right quadrant using a stainless steel needle fitted on a plastic syringe. The volume of dose formulation administered to each animal was adjusted according to the body weight. A constant dosage volume of 5 mL/kg was used. The exact dose administered to each animal was measured by weighing the dosing system before and after administration. The dose formulations was maintained under delivery conditions (at room temperature, protected from light) throughout the dosing

procedure. The test item dose formulations was stirred for at least 5 minutes before and throughout the dosing procedure.

Blood collection

Serial design:

At each scheduled sampling time, 200µL of blood was collected from the cannula on conscious rats. Blood was collected in 1.5 ml Eppendorf vial. After withdrawing the blood, the withdrawn volume was replaced with sterile saline solution (approx. 200µL). At the last sampling time of the dosing day, the cannula was flushing with an anticoagulant solution (heparinized saline solution) and was closed tightly. Blood was centrifuged at 10000g for 3 min at +4°C. Plasma samples (approximately 40 µL) was collected in a 96 well plate in triplicate and then stored at -20°C until bioanalysis.

Clinical observation

All animals were observed for mortality and general condition at least twice daily (once in the morning and once in the afternoon) during the week and once daily during the weekend. The adverse event, clinical signs or death were reported in the study book.

Bioanalytical Methods

• Pump : Thermo Accela

• Auto Sampler: Thermo Accela Open AS

• Column Oven : Agilent 1100 Heater

• Mass Spectrometer : Thermo TSQ Quantum Ultra

• Software : Excalibur version 2.1.0

Chromatography

• Column: Hypersil Gold C18 (50x2.1mm ; 1.9µm)

• Flow : 700µl/mn

• Oven temperature: 40°C

• Mobile phase :

Phase A aqueous-acidic: formic acid (0.1%) / HPLC water

Phase B organic-acidic: formic acid (0.1%) / Acetonitrile

Table S7 Gradient mode

**Time (min)**	0	0.1	0.5	1.5	1.6	2.0
**% Phase A**	95	95	5	5	95	95
**% Phase B**	5	5	95	95	5	5

Injection Volume: 5 µL

Mass spectrometry

• Ionization mode: ESI positive mode

• Capillary Temperature: 350 °C

• Vaporizer Temperature: 350 °C

Table S8 MS parameters.

Skimmer offset	(S)-CE-123: unused<br>Tolbutamide (ISTD): unused
Collision Energy Voltage	(S)-CE-123: 11 V<br>Tolbutamide (ISTD): 30 V
Multiple Reaction Monitoring	(S)-CE-123: m/z 314.045 → 167.060<br>Tolbutamide m/z 271.4 → 91.2
Retention Time	(S)-CE-123: 0.92mn<br>Tolbutamide: 0.93mn

Preparation of quality control and calibration samples

Quality Control (QC) samples and Calibration Samples wereprepared daily by spiking rat plasma or brain homogenates with working solutions prepared from independent weighing. The calibration curve werecalculated from calibration levels at 2.5, 5, 10, 25, 50, 100, 200, 500, 1000, 2000 and 5000 ng/mL (at least 75% of all standards used to make up the curve will back-calculate to ± 20% of their nominal value and at least six non-zero concentrations that back-calculate to ± 20% of their nominal value wereused for the quantitation of the samples). The concentrations for Quality Controls were 12.5, 50, 150, 450, 1500 and 4000 ng/mL (At least 66% of all QC samples must be within ± 20% of their nominal value and at least 50% of QC samples at a given concentration must be within +/- 20% of their nominal value). Regressions used were linear or quadratic regressions (plasma and brain, respectively) not forced through the origin, and weighted by $1/x^2$ or $1/x$ (plasma and brain, respectively) (with R2 >0.98). The lower limit of quantification (LLOQ) was defined as being less or equal to 120% of the area of the first EB following the ULOQ.

PK analysis

The pharmacokinetic parameters were calculated from the individual plasma concentrations using the program WinNonLin 7.0 (Phoenix 64), non-compartmental model 200.

Study Plan adherence and deviation

No deviation was reported during the study

Results

Table S9 Individual plasma concentrations of (S)-CE-123 (serial design)

Animal	1		2		3	
Animal Weight (g)	250		258		267	
Dose Volume (mL)	1.00		1.03		1.07	
Actual Dose (mg/kg)	10		10		10	
Nominal Sampling Timepoint (h)	Actual Time (h)	Conc. (ng/mL)	Actual Time (h)	Conc. (ng/mL)	Actual Time (h)	Conc. (ng/mL)
**0.25**	0.25	5295	0.25	9998	0.25	11591
**0.50**	0.50	3984	0.50	4836	0.50	8834
**1.00**	1.00	2238	1.00	2629	1.00	4878
**3.00**	3.00	260	3.00	464	3.00	1969
**7.00**	7.00	3.40	7.00	2.66	7.00	78.2
**24.00**	24.00	BLQ	24.00	BLQ	24.00	BLQ

Table S10 Individual plasma and brain concentrations of (S)-CE-123 (destructive design and terminal sampling time of serial design@ 24hrs)

Time h	Animal ID	Plasma ng/ml	Brain ng/g	Kp Br:Pl
**0.25**	**7**	8738	5600	0.64
	**8**	3797	3787	1.00
	**9**	4413	4956	1.12
	**MEAN**	**5649**	**4781**	**0.92**
	**SD**	**2692**	**919**	**0.25**
**0.5**	**10**	2637	2586	0.98
	**11**	575	605	1.05
	**12**	4124	3795	0.92
	**MEAN**	**2445**	**2329**	**0.98**
	**SD**	**1782**	**1611**	**0.07**
**1**	**13**	1242	1335	1.08
	**14**	646	601	0.93
	**15**	1182	1268	1.07
	**MEAN**	**1023**	**1068**	**1.03**
	**SD**	**328**	**406**	**0.08**
**3**	**16**	182	183	1.0
	**17**	145	124	0.9
	**18**	31.5	26.6	0.8
	**MEAN**	**120**	**111**	**0.90**
	**SD**	**78.5**	**79.1**	**0.09**
**7**	**19**	BLQ	BLQ	NC
	**20**	BLQ	BLQ	NC
	**21**	10.7	BLQ	NC
	**MEAN**	**BLQ**	**BLQ**	**NC**
	**SD**	**-**	**-**	**-**
**24 (brain serial)**	**1**	BLQ	BLQ	NC
	**2**	BLQ	BLQ	NC
	**3**	BLQ	BLQ	NC
	**MEAN**	**BLQ**	**BLQ**	**NC**
	**SD**	**-**	**-**	**-**
				**0.96**
				**0.06**

Table S11 Individual plasma pharmacokinetics parameters of S-CE-123.

PK Parameter	IP 10 mg/kg		
	1	2	3
Dose (mg/kg)	10	10	10
Dose (µmol/kg)	32	32	32
C_0 / C_{max} (ng/mL)	5295	9998	11591
C_0 / C_{max} (nM)	16838	31796	36862
T_{max} (h)	0.25	0.25	0.25
$t_{1/2}$ (h)	0.64	0.59	0.98
MRT (h)	-	-	-
Vdss (L/kg)	-	-	-
CL / CL_F (mL/min/kg)	31	22	10
AUC_{inf} (ng.hr/mL)	5406	7692	16187
AUC_{inf} (nM.hr)	17191	24463	51475
AUC_{0-t} (ng.hr/mL)	5403	7690	16076
AUC_{0-t} (nM.hr)	17181	24456	51123
Fraction Absorbed	-	-	-
C_{last} (ng/mL)	3.4	2.7	78.2
Number of Points used for Lambda z	3	3	3
AUC % Extrapolation to infinity	0.1	0.0	0.7
T_{last} (h)	7	7	7
